# Highly Unsaturated Lignin Through Clean and Efficient Ru‐Catalyzed Glycerol Carbonate Allylation and Ring‐Opening Reaction Strategies

**DOI:** 10.1002/cssc.71047

**Published:** 2026-09-22

**Authors:** Claudie Simon, Tiphaine Richard, Sofiane Guessasma, Mathieu Sauthier, Klara Jastak, Clément Dumont

**Affiliations:** ^1^ Univ. Lille, CNRS, Centrale Lille, ENSCL, Univ. Artois, UMR 8181‐UCCS ‐ Unité de Catalyse et Chimie du Solide Villeneuve d'Ascq France; ^2^ Icam School of Engineering, Lille Campus Lille Cedex France; ^3^ INRAE, Research Unit BIA UR1268 Nantes France

**Keywords:** allylation, biomass, catalysis, lignin functionalization, ring‐opening

## Abstract

Innovative lignin functionalization strategies which graft a large amount of unsaturation onto lignin have been developed. Ruthenium catalysis using allyl alcohol or diallyl ether has been used to develop ready‐to‐graft allylic synthons based on glycerol carbonate and related derivatives. Allylated glycerol carbonate and glycidyl allyl ethers were thus used in effective ring‐opening reactions with high atom economy to convert Kraft, Organosolv, and Soda lignins without prior purification. Glycidyl allyl ethers were grafted onto lignin with a substitution degree of hydroxyl groups up to 90%. Technical and modified lignins were characterized by nuclear magnetic resonance (NMR), infrared (IR), size‐exclusion chromatography (SEC), and thermal properties were determined by differential scanning calorimetry (DSC). The grafting efficiency produces modified lignins with a high level of unsaturation, with thermal properties allowing material shaping, providing new opportunities for material applications.

## Graphical abstract

An innovative, ruthenium‐catalyzed strategy successfully functionalizes unpurified technical lignins with ready‐to‐graft allylic synthons derived from glycerol carbonate. This atom‐economical ring‐opening reaction achieves up to 90% hydroxyl substitution. The resulting highly unsaturated lignins display excellent thermal and shapable properties, unlocking new opportunities for sustainable, high‐performance material applications.

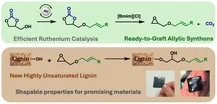

## 1 Introduction

In light of environmental concerns and the global need for more sustainable practices, the development of bio‐based alternatives to fossil‐based materials has become a major challenge. The increasing demand for renewable resources has highlighted lignocellulosic biomass, an abundant and renewable source of carbon, as a promising alternative. Lignin is one of its main components and is clearly under‐exploited despite being one of the most abundant natural polymers on Earth [1, 2]. Lignin represents around 20% of lignocellulosic biomass. It is biosynthesized from phenolic compounds, making this aromatic structure abundant in the lignin scaffold and attractive for many potential alternatives in the plastics and resins industry [3]. 100 million tons of lignin are estimated to be extracted annually as a byproduct of the paper industry [4]. However, despite the industrial potential, most of the lignin is used as an energy source, with 98% burned for energy production [5]. The valorization of lignin as a material remains challenging due to poor mechanical properties. Nevertheless, lignin shows notable UV‐shielding, fire‐retardant, antioxidant, hydrophobic, and antimicrobial properties [6, 7]. The chemical functionalization of lignin offers a major opportunity to adjust mechanical limitations and shape the reactivity of this material, providing a pathway for a wide range of high‐value‐added applications [8, 9]. Esterification and etherification are the most commonly used methods to modify lignin [8, 9, 10]. Esterification can be performed directly on the aliphatic hydroxyl groups of lignin using carboxylic acids [11]. However, the conversion of phenolic groups, abundant in lignin, requires activated reagents such as acyl chlorides [12, 13] or using the Steglich reaction with DCC/DMAP [14, 15]. Thus, maximum esterification of lignin hydroxyl groups leads to the production of salts or coproducts in stoichiometric quantities. To efficiently convert phenolic hydroxyl groups in lignin, etherification reactions provide interesting alternatives. Ether bonds increase the resistance to hydrolysis, an essential property for sustainable material applications. These transformations can be achieved through Williamson‐type reactions [16, 17], epoxide [18, 19, 20, 21], or carbonate ring‐opening transformations [22].

Among etherification strategies, lignin allylation is particularly attractive, as allylated lignin can be used as a macromonomer in a wide range of polymerization reactions [23]. The introduced unsaturations provide a versatile platform for the development of thiolene reactions [24, 25], metathesis [26], Claisen rearrangement [27], radical polymerization [28], and even for the synthesis of epichlorohydrin‐free epoxy resins [29, 30]. This polyfunctionality represents a remarkable opportunity for the creation of high‐performance bio‐based materials. However, conventional lignin allylation protocols have important drawbacks. These processes rely on the use of allyl halides under strongly basic conditions, generating stoichiometric amounts of inorganic salts and producing toxic halogenated waste [3, 31]. In addition, they typically use petrochemical reagents and toxic solvents, which are inconsistent with the principles of green chemistry despite the use of a renewable resource. For functionalization in line with sustainable development, ecofriendly alternatives are emerging [32]. Diallyl carbonate, an environmentally friendly reagent, enables solvent‐free allylation of lignin [33, 34, 35]. This still faces the disadvantage of requiring a stoichiometric amount of base to activate the lignin. More advanced catalysis approaches have emerged to overcome these limitations [36]. Palladium‐catalyzed Tsuji‐Trost reactions [37] or zirconium [38, 39] allylation reactions are used to graft the C3 allyl moiety, while the palladium‐catalyzed butadiene telomerization allows the grafting of C8 octa‐2,7‐dien‐1‐yl tails onto lignin [40]. These approaches currently require high catalyst loadings to convert lignin due to its impurities and recalcitrance. Furthermore, metal traces may remain in the final product, which is a hindrance for some material applications.

The search for strong, efficient, affordable, and clean ways of modifying lignin is driving research routes towards combining catalytic reactions to synthesize suitable reactive synthons with ring‐opening transformation to cleanly graft lignin. This approach allows the catalyst to be removed from the molecular reagent before grafting onto the lignin, enabling metal recyclability. Glycerol carbonate (GC) is an attractive starting material for the synthesis of such a synthon. GC can be obtained from the reaction between CO_2_ or dimethyl carbonate and glycerol, a renewable resource [41, 42]. Glycerol carbonate can be used under relatively mild conditions to access etherified lignin [43]. This two‐step grafting strategy has been used in our laboratory to introduce octa‐2,7‐dien‐1‐yl onto lignin using nickel catalysis to etherify GC [44]. However, the two unsaturations per chain grafted following this technique are not equivalent (allylic and terminal), which can complicate the subsequent reactions for material applications. In addition, the reaction used requires the use of butadiene gas and pressurized systems which could be an obstacle to scaling up. This article proposes using a ruthenium catalyst to synthesize allylated glycerol carbonate (AGC) and ultimately introduce a maximum of the allyl moiety into the lignin. Ruthenium catalysts have been shown to be effective for the preparation of allyl ethers from phenols [45, 46] and 1‐octanol [47]. This ruthenium catalysis is typically performed under mild conditions and is easily upscalable, without the need for pressurized equipment.

## 2 Materials and Method

### 2.1 General Considerations

Kraft lignin was provided by Centre technique du Papier (CTP), Organosolv by ChemicalPoint, and Soda by GreenValue Enterprises LLC. Prior to use, all lignins were dried under vacuum at 50 °C for 48 h. Chemicals were purchased from Aldrich, TCI, Alfa Aesar, Acros, and Strem. Tetrahydrofuran was purified using an MBraun SPS‐800 solvent purification system. PA12 corresponds to Vestamid X7167 from Evonik.

Thermal transitions and heat flows of the studied material were considered through differential scanning calorimetry (DSC) analyses. These were performed using a PerkinElmer DSC 4000 differential scanning calorimeter. Measurements were conducted at a heating rate of 10 K min^−1^ over a temperature range of 243–453 K, under a nitrogen flow (20 ml min^−1^).

Nuclear magnetic resonance (NMR) spectroscopy was used to characterize the structure and chemistry of lignin including functional group identification and monitor chemical modifications. NMR spectra were recorded in CDCl_3_ (99.8% atom D), purchased from Aldrich. ^1^H NMR spectra were obtained at either 300 or 400 MHz, and ^13^C were recorded at 75 MHz with ^1^H decoupling and 101 MHz with both ^1^H and ^31^P decouplings; and ^31^P spectra were acquired at 121 and 162 MHz with ^1^H decoupling. Chemical shifts (^1^H and ^13^C) were referenced in parts per million relative to TMS (*δ* = 0.00 ppm) and calibrated using the residual solvent peak (^1^H, CDCl_3_ 7.26 ppm).

Infrared (IR) spectroscopy was used to identify chemical bonds and functional group modification in the studied materials. IR spectra were recorded using a PerkinElmer Spectrum Two FT‐IR spectrometer (model L1600401) equipped with a MIR TGS detector, covering the spectral range of 7500–550 cm^−1^. Measurements were carried out in attenuated total reflectance (ATR). Typically, 4 scans were accumulated for each spectrum (resolution of 4 cm^−1^).

In order to access the variability of the polymer size and chain length, the number‐average molecular weight (*M*_n_), weight‐average molecular weight (*M*_w_), and dispersity (*Đ*) of the product were determined by size‐exclusion chromatography (SEC) in tetrahydrofuran at 30 °C, with a flow rate of 0.6 mL min^−1^. The sample concentration was 0.50 wt%. *M*_n_, *M*_w_, and *Đ* were calculated from the refractive index (RI) signal using a calibration curve based on polystyrene (PS) standards from Polymer Standards Service. Analyses were performed on a ThermoScientific—Dionex UltiMate 3000 RS apparatus equipped with Tosoh TSKgel columns SuperH2000, SuperH3000, and SuperH5000.

### 2.2 Titration of the Hydroxyl Groups in the Technical Lignins and Modified Lignins

^31^P NMR experiments were performed following the method described by Granata and Argyropoulos [48]. Lignin (30 mg) was dissolved in a CDCl_3_/pyridine mixture (1:1.6 v/v, 0.5 mL). The phosphitylation reagent, 2‐chloro‐4,4,5,5‐tetramethyl‐1,3,2‐dioxaphospholane (150 μL), was added, followed by the internal standard, N‐hydroxy‐6‐norbornene‐2,3‐dicarboximide (100 μL of a 0.1 M solution in the same CDCl_3_/pyridine mixture). To facilitate homogenization and enhance phosphorus relaxation, chromium (III) acetylacetonate (100 μL of a 0.014 M solution in the same solvent mixture) was added. Spectra were acquired with a 15 s relaxation delay and averaged over 512 scans. Chemical shifts were referenced to the signal of the phospholane hydrolysis product at 132.2 ppm. The quantification of functional groups was based on the integrated signal of the internal standard. The degree of substitution of hydroxyl groups was calculated by comparing the functional group content of the technical lignin with that of the modified lignin (see Figure S13 for details).

### 2.3 General Procedure of Allylation of Glycerol Carbonate (AGC)

Dried and degassed GC (0.45 mL, 5.3 mmol) was added into a Schlenk tube under an inert atmosphere containing p‐toluenesulfonic acid (PTSA), AgOTs, and RuCp(PPh_3_)_2_Cl. Degassed THF was added, and the Schlenk tube was heated at 40 °C for 5 min under magnetic stirring. Diallyl ether (DAE) was added, and the Schlenk tube was heated at 60 °C for 3 h. After the reaction, the Schlenk tube was cooled to room temperature. The isolated product was obtained by column chromatography on silica gel with petroleum ether/ethyl acetate 70:30. The product was obtained as a yellowish liquid with a 78% yield.

### 2.4 Procedure of Telomerization of Glycerol Carbonate (GC)

GC (freezed pump) (5 g, 42.3 mmol, 1 eq.), Ni(cod)_2_ (196 mg, 713 µmol, 1.5%), and dppb (460 mg, 1079 µmol, 2.25%) were gathered in an autoclave in a glove box. Then, condensed 1,3‐butadiene (20 mL, 229 mmol, 5 eq.) at −50 °C was inserted in the autoclave under nitrogen, which was then placed at 80 °C for 16 h. After the reaction, the reactor was cooled to room temperature, and the excess of gas was vented. The product was isolated by column chromatography on silica gel with petroleum ether/ethyl acetate 80:20. 71% isolated yield GCT (38% branched–62% linear) [44].

### 2.5 Procedure of Lignin Grafting Using Glycerol Carbonate (L‐AGC)

Lignin (0.5 g, 3.02 mmol/g OH), AGC (6 eq.), and base K_2_CO_3_ (0.1 eq.) were gathered in a Schlenk tube, and DMSO (9 eq.) was added. The reaction was heated at 170 °C during 3 h. After the reaction, the Schlenk tube was cooled, and modified lignin was precipitated 3 times using a water‐diethyl ether and petroleum ether (1:1:1) mixture. The modified lignin was dried under vacuum at 40 °C overnight.

### 2.6 Procedure for the Conversion of Modified Glycerol Carbonate into the Corresponding Epoxide (Decarbonation Reaction)

AGC (5 g) and the catalyst 1‐butyl‐3‐methylimidazolium chloride [Bmim][Cl] (2.5 mol%) were added in a Schlenk tube mounted with a microdistillation system cooled with a water condenser. The system is then heated at 170 °C for 48 h (see Figure S16). The product GAE was obtained as a yellowish liquid with an 85% yield.

GCT follows the same procedure, except that the system was under vacuum and the collecting Schlenk was kept in liquid nitrogen. A yield of 65% was achieved. 16% of GCT remains in the final product. The obtained GTE product contained 94% of linear and 6% of branched isomers.

### 2.7 General Procedure of Lignin Grafting Using Glycidyl Allyl Ether (L‐GAE) and Glycidyl Telomer Ether (L‐GTE)

Lignin (0.25 g), the glycidyl ether (3.5 eq./lignin OH), and K_2_CO_3_ (0.05 eq.) were added in a Schlenk tube and dissolved in dioxane (0.5 mL) was added. The reaction was heated at 150 °C during the time chosen. After the reaction, the Schlenk tube was cooled, and modified lignin was precipitated 3 times by petroleum ether. The modified lignin was dried under vacuum at 40 °C overnight.

### 2.8 Extrusion of PA12–Lignin Filaments, and 3D Printing

Extrusion of the synthesized materials was conducted with ThermoFisher Process 11 twin‐screw extruder (Thermo Fisher Scientific, Waltham, Massachusetts, USA) with a screw speed of 100 rpm and a 200 g/h feed rate. The barrel temperature profile across the eight zones was set to 100, 170, 180, 190, 200, 210, 215, and 215 °C, respectively.

The composite filament was produced using an Artme3D MK2.5 filament extruder (2024 model). The filament was produced using a single‐screw extruder (2:1 compression ratio) at 227 °C and a screw speed of 12 rpm. The extrudate was cooled at 12% fan capacity and drawn with a 4 g take‐up force at a 17 cm distance from the die. Ambient temperature was maintained at 21 °C. The feedstock consisted of precompounded PA12 blended with 10% of modified lignin and ground into pellets prior to extrusion.

The specimens were fabricated using a Volumic STREAM 30 Ultra fused filament fabrication (FFF) 3D printer, with a 0.4 mm nozzle diameter. Printing was performed with a printing temperature of 245 °C and a heated bed set between 80 and 90 °C. The layer height was set to 0.2 mm, with a maximum print speed of 30 mm/s.

## 3 Results and Discussion

Ruthenium catalysis using the RuCp(PPh_3_)_2_Cl precursor provided an efficient pathway for grafting allyl groups onto phenols and aliphatic hydroxyl groups. This ruthenium catalysis is typically performed under mild conditions and is easily upscalable. This allylation reaction uses allyl alcohol or Diallyl ether (DAE) as reagents and does not generate stoichiometric salts as side products. Based on the previous results, the reaction was performed with Glycerol carbonate (GC) and allyl alcohol as the allylating agent and in the presence of 0.10 mol% RuCp(PPh_3_)_2_Cl and 10 mol% PTSA in THF at 60 °C during 3 h. 0.2 mol% AgOTs was also added to activate the catalytic precursor by removing the chloride. Under these conditions, Allylated Glycerol Carbonate (AGC) is obtained with up to a 42% yield. This reaction with allyl alcohol proved to be limited in yield. However, the use of DAE in place of allyl alcohol showed more promising results. An optimization study was therefore conducted with this reagent to improve allylation yields (Table 1). The kinetics of the reaction were first studied. Increasing the reaction time enhances the reaction yield from 54% to 70%. (Table 1, compare entries 1–3). The introduction of a larger amount of the DAE reagent, from 2 to 4 equivalents (entries 3 and 4), allowed the yield to improve from 70% to 75%. However, a further increase does not result in a significant change in yield. Surprisingly, the removal of AgOTs from the catalytic cycle has not had any impact on the efficiency of catalysis (compare entries 4 and 6). This is probably balanced by the excess of PTSA used for the reaction. Nevertheless, this is an advantage to simplify purification protocols and reduce the costs of the reaction. Limiting the volume of the solvent has a negative impact on yield. Indeed, the gradual reduction in the amount of solvent, from 10 to 5 mL, reduced the yield from 73% to 66%. The yield dropped to 43% with 1 mL of THF (compare entries 6–8). Increasing the temperature from 60 to 90 °C increases the yield from 66% to 78% (compare entries 7 and 9). Furthermore, raising it to 110 °C showed no further improvement (entry 10). It should be noted that the reaction remains efficient at lower catalytic loadings. The use of 0.05 mol% ruthenium afforded a higher yield of 71% (entry 11). In addition, reducing the amount of PTSA leads to a less efficiency. The yield was dropped from 78% to 67% yield with, respectively, 10 and 5 mol% of p‐TSA (compare entries 9 and 12). The AGC was finally isolated by silica gel column chromatography with a 75% yield from the crude obtained with the optimized reaction conditions (entry 9). HRMS, ^1^H, and ^13^C NMR of the compound are available in (Figures S1 and S2). This purified product was then used as a linker for grafting lignin.

**TABLE 1:** Optimization of catalytic reaction conditions for AGC synthesis.

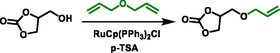							
Entry	[Ru], %mol	AgOTs, %mol	*t*, h	DAE, eq	THF, mL	*T*, °C	Yield^a^
1	0.1	0.2	1	2	10	60	54%
2	0.1	0.2	3	2	10	60	67%
3	0.1	0.2	6	2	10	60	70%
4	0.1	0.2	3	4	10	60	75%
5	0.1	0.2	3	6	10	60	76%
6	0.1	—	3	4	10	60	74%
7	0.1	—	3	4	5	60	66%
8	0.1	—	3	4	1	60	43%
9	0.1	—	3	4	5	90	78%
10	0.1	—	3	4	5	110	78%
11	0.05	—	3	4	5	90	71%
12^b^	0.1	—	3	4	5	90	67%

*Note* *:* Conditions: 0.45 mL (5.3 mmol) glycerol carbonate, THF, [Ru] = RuCp(PPh_3_)_2_Cl, p‐TSA = 10 %mol, N_2_ atmosphere, Schlenk equipped with a Rotaflow stopcock.

a Yield determined by ^1^H NMR (see Figure S3).

b p‐TSA = 5 %mol.

**SCHEME 1:**
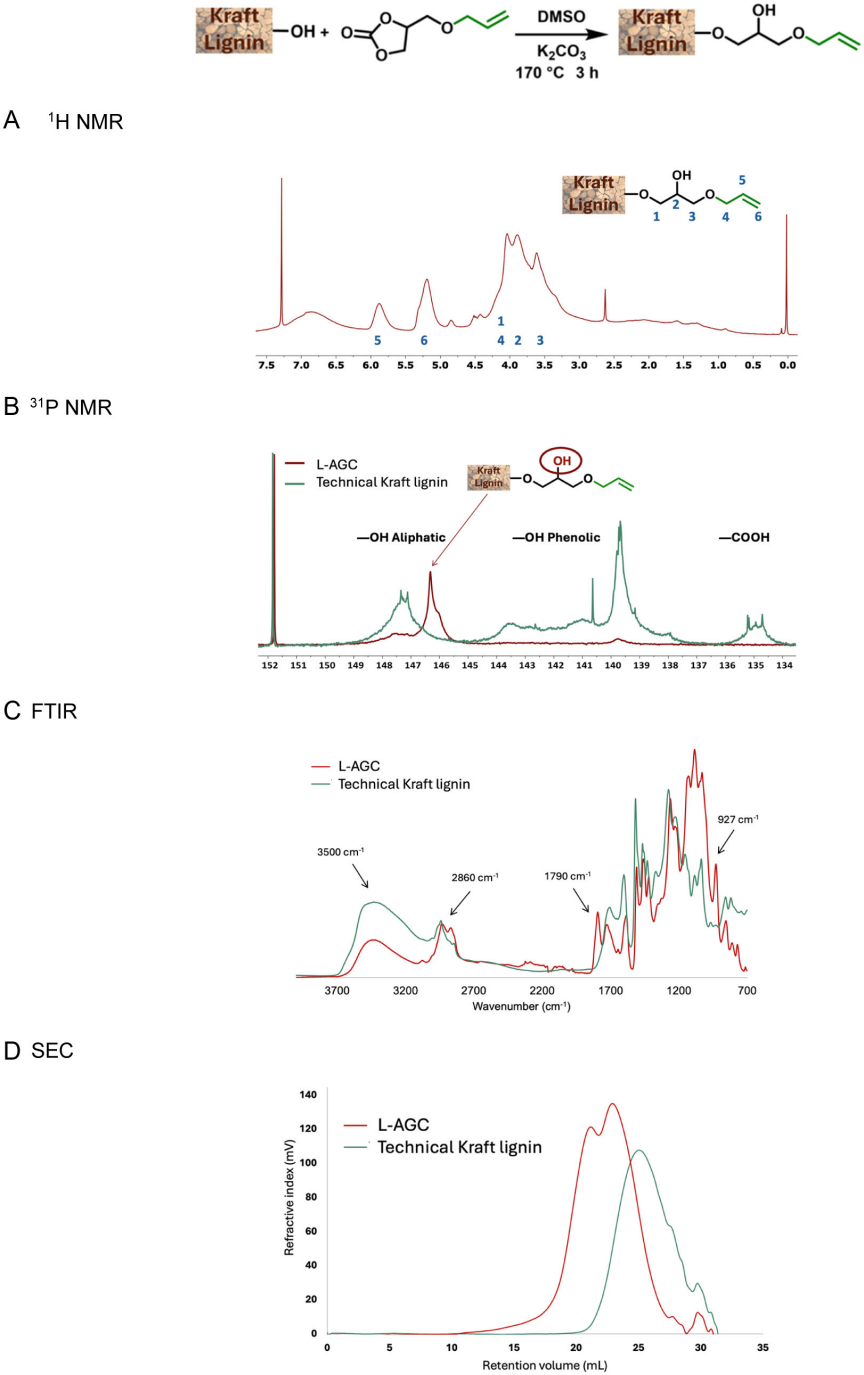
Grafting of AGC on Kraft lignin – (A) ^1^H NMR spectra of AGC on lignin (L‐AGC) in CDCl_3_ – (B) ^31^P RMN spectra of technical Kraft lignin and L‐AGC – (C) FTIR spectra of technical Kraft lignin and L‐AGC – (D) comparison of SEC spectra of technical Kraft lignin and L‐AGC.

AGC was first grafted onto Kraft lignin following the protocol used in our previous work [44]. 0.5 g of Kraft lignin was diluted in 2 mL of DMSO and reacted with 6 equivalents of AGC with respect to the alcohols (aliphatic and phenolic) of the lignin, at a temperature of 170 °C during 3 h and in the presence of 0.1 equivalents of K_2_CO_3_ (Scheme 1). ^1^H NMR (Scheme 1A) of the functionalized lignin gives evidence of an efficient grafting protocol. The spectrum shows characteristic signals of technical Kraft lignin, a broad singlet at 3.0–4.0 ppm for the O=CH_3_ groups, and a broad signal at 6.2–7.5 ppm that accounts for the aromatic protons. Additional large signals between 5.0‐5.5 and 5.75‐6.25 ppm are, respectively, attributed to the CH_2_ and CH protons of the allylic double bond. The protons of the glycerol and the O=CH_2_ of the allyl moiety are all located between 3.25 and 4.25 ppm. These signals are superposed with the signal attributed to the methoxy groups of the lignin. The presence of these protons is clearly observed in the 2D NMR correlation (^13^C spectra and HSQC are available in Figures S8 and S9). Weak signals at 4.5 and 4.8 ppm correspond to the protons of the cyclic carbonate of the unreacted AGC reagent. The grafting was further assessed by ^31^P NMR spectroscopy (Scheme 1B). The comparison of the NMR spectra obtained for the phosphorylated functionalized lignin and phosphorylated Kraft lignin evidences a large disappearance of signals between 137‐144 and 134‐136 ppm corresponding, respectively, to phenolic hydroxyl groups and carboxylic acid groups. Additionally, the signals that correspond to the aliphatic hydroxyl groups in the native lignin (146.5–149.0 ppm) are less intense, and new signals attributed to the new alcohols formed after the ring‐opening reaction are visible between 145.5 and 147.0 ppm. Quantification calculations (see Section S13) show that 68% of the total hydroxyl groups have been substituted (9% of the aliphatic groups, 84% of the phenolic groups, and 100% of the carboxylic acids). These analyses confirm the opening reaction of cyclic carbonates on phenols and COOH and the addition of new functionalities on lignin (unsaturation and aliphatic alcohol) in accordance with the results previously published [44].

The amount of total hydroxyl groups available in the modified lignin is lower than in the Kraft lignin, decreasing from 7 to 3.8 mmol/g. This is caused by two factors: the decrease in the mass of lignin in the sample (with the added chain weights) and the conversion of some of the hydroxyl groups by transcarbonation. These secondary reactions, known for ring opening with carbonates, are clearly visible by FTIR spectroscopy with the appearance of a new band at 1790 cm^−1^ which is characteristic of carbonyl groups (Scheme 1C). Moreover, this analysis also confirms the modification of lignin with higher band intensities at around 1100 cm^−1^, this observation is consistent with the presence of new alkane chains after the grafting reaction. In addition, the signal at 927 cm^−1^ is assigned to alkene sp_2_ C=H bending patterns. The reduction in the number of hydroxyl groups is shown by a decrease in the OH stretching band at 3500 cm^−1^, also suggesting the presence of transcarbonation. These can also be observed with SEC analyses of modified lignins. The increase in molar mass on the product (*M*_n_ = 4200 g/mol, *Đ *= 2.6) compared to the technical lignin (*M*_n_ = 650 g/mol, *Đ *=  3) provides information on the significant amounts of additional chains added to the lignin. However, compared to technical lignin, the L‐AGC curve is clearly bimodal, confirming the existence of cross‐linking via transcarbonation. Thermal analysis by DSC indicates a glass‐transition temperature for L‐AGC of 94 °C, showing the impact of the grafted chains, as the technical lignin has a much higher initial *T*_g_ of 155 °C.

Thus, the grafting of a large number of allylic unsaturated chains onto lignin is efficient in two steps from GC using a clean, salt‐free ruthenium catalytic reaction. Despite the efficiency of this grafting method, the presence of cyclic carbonates leads to side reactions such as transcarbonation, which results in reticulated lignin, thus limiting its potential. It should also be noted that purification of lignin is more difficult due to the high boiling point of the grafting reagent that prevents any removal with a distillation protocol and the use of DMSO as the solvent of reaction, which is also ultimately difficult to remove. To avoid these limitations, the decarbonation of AGC to access the corresponding epoxide derivative, Glycidyl Allyl Ether (GAE), has been done (Table 2). The epoxide analog offers several advantages over the carbonate. Its boiling point is significantly lower, and its slightly reduced polarity enhances solubility in common solvents. Moreover, the epoxide exhibits higher reactivity toward ring opening, potentially enabling reactions at milder temperatures. Under such conditions, volatile and easily removable solvents could be employed as alternatives to DMSO. Finally, the absence of a carbonate group prevents undesired transcarbonation and cross‐linking side reactions. The reaction protocol is based on studies in the literature, in particular, on the transformation of 1,2‐butylene carbonate [49]. Under the effect of temperature and in the presence of an imidazolium salt (here 2.5 mol% of [Bmim][Cl]), the cyclic carbonate releases CO_2_ and is converted into the epoxide GAE (Table 2). A yield of 85% of GAE is obtained after 48 h of reaction at 170 °C. The structure of the product was confirmed by ^1^H and ^13^C NMR see, Figures S14 and S15).

**TABLE 2:** Optimization of AGC decarboxylation by continuous step‐up into GAE.

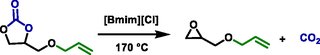			
Entry	*T*, °C	Time, h	Yield, %
1	170	5	34
2	170	24	57
3	170	48	85

*Note* *:* Conditions: 5 g of glycerol carbonate, 2,5 mol% butyl methyl imidazolium chloride.

This step has the advantage of being performed without purification of the AGC. This allows the AGC to be converted into GAE and purified by distillation in a single step. Thus, the ruthenium used during the allylation reaction can be easily recovered for potential recycling. This also ensures the absence of any metal traces in the final lignin‐based material during the grafting of GAE.

**FIGURE 1:**
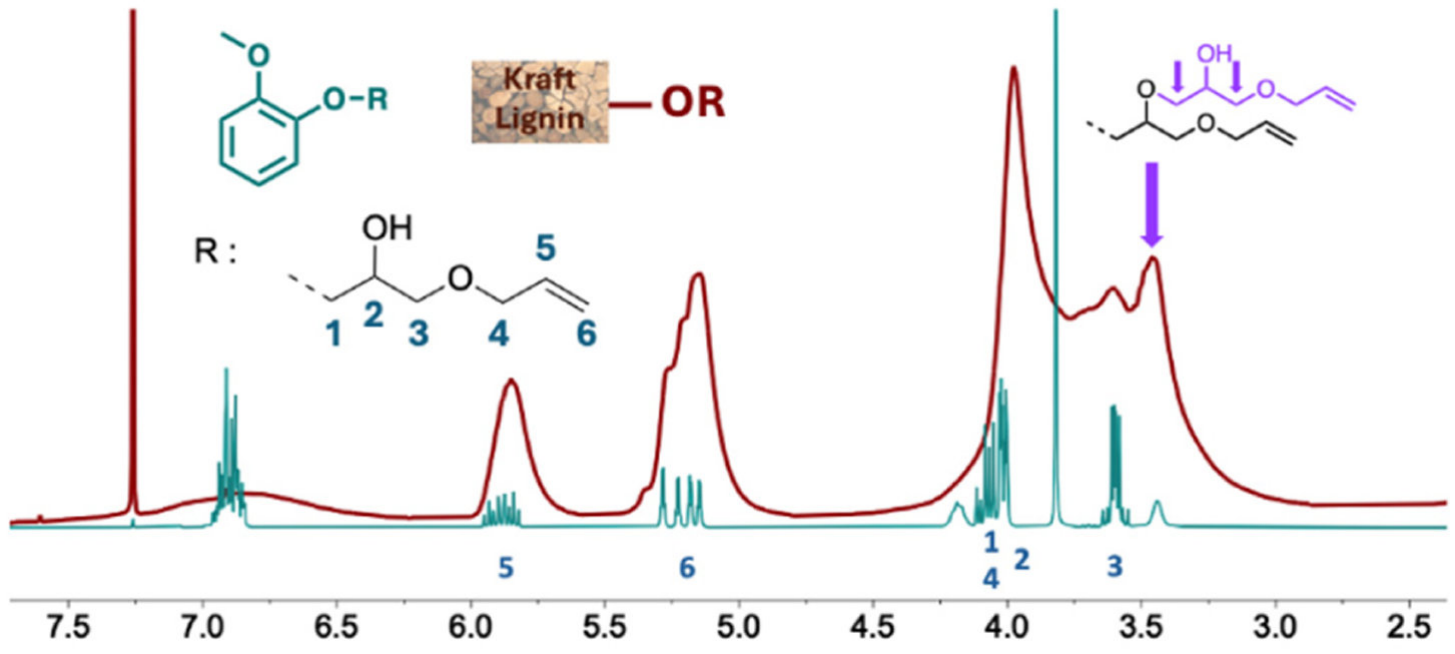
Comparison of ^1^H NMR of model molecule (green) and L‐GAE (red).

The GAE molecule thus obtained was involved in a ring‐opening reaction with Kraft lignin (Table 3). The transformation was carried out in a closed reactor with dioxane, selected for easy removability, in the presence of 0.1 equiv. of K_2_CO_3_ at 150 °C for 3 h. ^31^P NMR analysis of the L‐GAE‐modified lignin shows a profile similar to the L‐AGC spectrum (see Section S21). Quantification indicates a degree of substitution of 90% of total lignin hydroxyl groups, with complete conversion of both phenols and carboxylic acids and 57% of aliphatic alcohols. Thus, compared to the reaction that involves a carbonate, the reaction with an epoxide shows greater reactivity at lower temperatures and converts a higher proportion of aliphatic hydroxyl groups. The same reaction was performed with guaiacol as a model molecule of lignin (see Sections S17 and S18 for product information). The comparison of the ^1^H NMR spectra obtained with the functionalized lignin L‐GAE and the guaiacol derivative shows a good correlation between the signals observed (Figure 1). The absence of signals around 4.5 ppm in the ^1^H NMR spectrum of L‐AGC, which would correspond to the alternative regioisomer arising from nucleophilic attack at the internal carbon of the epoxide ring, confirms the selective ring‐opening pathway. This outcome is further supported by comparative 2D HSQC and ^13^C NMR analyses of the modified lignins (see Sections S19 and S20). However, an additional signal was observed at 3.4 ppm in the ^1^H NMR spectrum of L‐AGC, which does not appear in the spectrum of the guaiacol model and is insignificant in the case of L‐AGC. This signal is attributed to O–CH_2_ protons formed after the ring‐opening reaction on an aliphatic alcohol (Figure 1—proton 1 on an aliphatic alcohol and relevant with HSQC) [50]. However, this signal appears to be over‐represented with regard to the quantity of aliphatic hydroxyl groups available on lignin. The ^1^H NMR quantifications carried out on the allylic signals (Figure 1 ‐ Signals 5 and 6) also revealed a quantity of unsaturation higher than the initial OH content of the lignin. A control reaction without lignin was performed and did not show significant polymerization of the epoxide GAE under these reaction conditions (see Figure S24). Considering the conversion on the aliphatic alcohols of the lignin, the main hypothesis appears to be that GAE also reacts on the new aliphatic OH groups formed after the ring‐opening reaction with phenols (see Figure 1—purple scheme). Based on the ^31^P NMR results and considering that each ring‐opening reaction replaces one lignin OH group with a single new one, the lignin mass percentage in the sample can be determined (see Table 3, “Lignin wt%”). The excess mass corresponds to the grafted synthon, which can be quantified to estimate the overall number of GAE ring‐opening events per initial lignin OH group (see Table 3, grafted chains per OH). All the calculation explanations are available in the Supporting Information (see Section S13).

**TABLE 3:** Optimization of catalytic reaction conditions for glycidyl allyl ether (GAE) grafting on lignin.

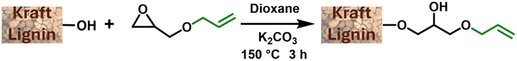											
Entry	GAE, eq	K_2_CO_3,_ eq	*T*, °C	Time, h	Dioxane, mL	Substitution degree, %^a^					
						OH tot.	OH Al.	OH Ph.	COOH	Lignin wt%	Grafted chains per OH
1	3.5	0.1	150	3	0.5	90	57	100	100	45	1.6
2	3.5	0.05	150	3	0.5	90	55	100	100	47	1.4
3	3.5	0.025	150	3	0.5	62	<1	79	100	67	0.6
4	1.8	0.05	150	3	0.5	88	49	100	100	49	1.3
5	3.5	0.05	125	3	0.5	78	3	100	100	62	0.8
6	3.5	0.05	110	3	0.5	74	0	95	100	61	0.8
7	3.5	0.05	150	6	0.5	85	36	100	100	43	1.6
8	3.5	0.05	150	16	0.5	88	49	100	100	44	1.6
9	5.3	0.05	150	3	—	94	73	100	100	42	1.7

*Note* *:* Conditions: 0.250 g of Kraft Lignin, closed Schlenk equipped with a Rotaflow stopcock.

a Determined by ^31^P NMR.

Under the initial experimental conditions, the weight % of technical lignin in the sample was 45%, with 1.6 allylic chains per OH (Table 3, entry 1). These conditions, therefore, seem to promote multiple epoxide openings on the lignin. With the aim of reducing this rate of cycle overopening, the parameters were optimized, starting with the catalytic amount of base K_2_CO_3_ used in the reaction. The use of 0.05 equivalents of base shows similar substitution results with 90% total OH conversion. However, under these conditions, the rate of multiple epoxide opening is limited to 1.4 (Table 3 compares entries 1 and 2). Further reduction of the catalyst reduces the grafting efficiency on lignin to 62% of total OH (entry 3).

It is worth mentioning that under these conditions, the aliphatic hydroxyl groups of the lignin are not substituted, and the number of grafted chains is, as expected for 62% of conversion, 0.6. Furthermore, the ^1^H NMR of this product did not show an additional signal at 3.4 ppm (see Section S25). Interestingly, halving the amount of GAE maintains the high substitution rates, but without reducing the additional epoxide‐opening reactions, which are maintained at 1.3 (compare entries 2 and 4). Lowering the temperature to 125 °C is a suitable solution for reducing overopening. At this temperature the number of grafted chains per OH is lowered to 0.8; this value corresponds to the lignin substitution degree of 78% (entry 5). The reaction was also effective at 110 °C. At this temperature, up to 74% of the hydroxyl groups were converted, with an uncomplete conversion of the phenolic hydroxyl groups (entry 6).

The reduction in reaction temperature further elucidates the regioselective profile of the epoxide ring opening. As expected, even under slowed kinetic conditions, COOH groups are quantitatively substituted, underscoring the inherent chemoselectivity of the epoxide ring‐opening reaction with carboxylic acids. In contrast, aliphatic hydroxyl groups become completely unreactive at lower temperatures, confirming that they possess the lowest nucleophilic reactivity and are functionalized last among lignin’s hydroxyl moieties. Phenolic OH conversion is slightly attenuated under these milder conditions, placing their reactivity intermediate between acidic and aliphatic hydroxyl groups. In addition, comparative ^31^P NMR analysis (entries 2 and 3) shows no apparent selectivity between the phenolic subunits, as the H, G, and S‐condensed groups are functionalized uniformly.

Increasing the reaction time of the GAE grafting on lignin from 3 to 16 h did not show a significant influence on lignin substitution degree and only slightly increased the number of chains per OH (compare entries 2, 7, and 8). Solvent‐free transformation was also achieved, by slightly increasing the amount of GAE equivalent to 5.3 to ensure good reaction mixing (entry 9). The obtained substitution degree was very high and reached 94% of total OH.

**FIGURE 2:**
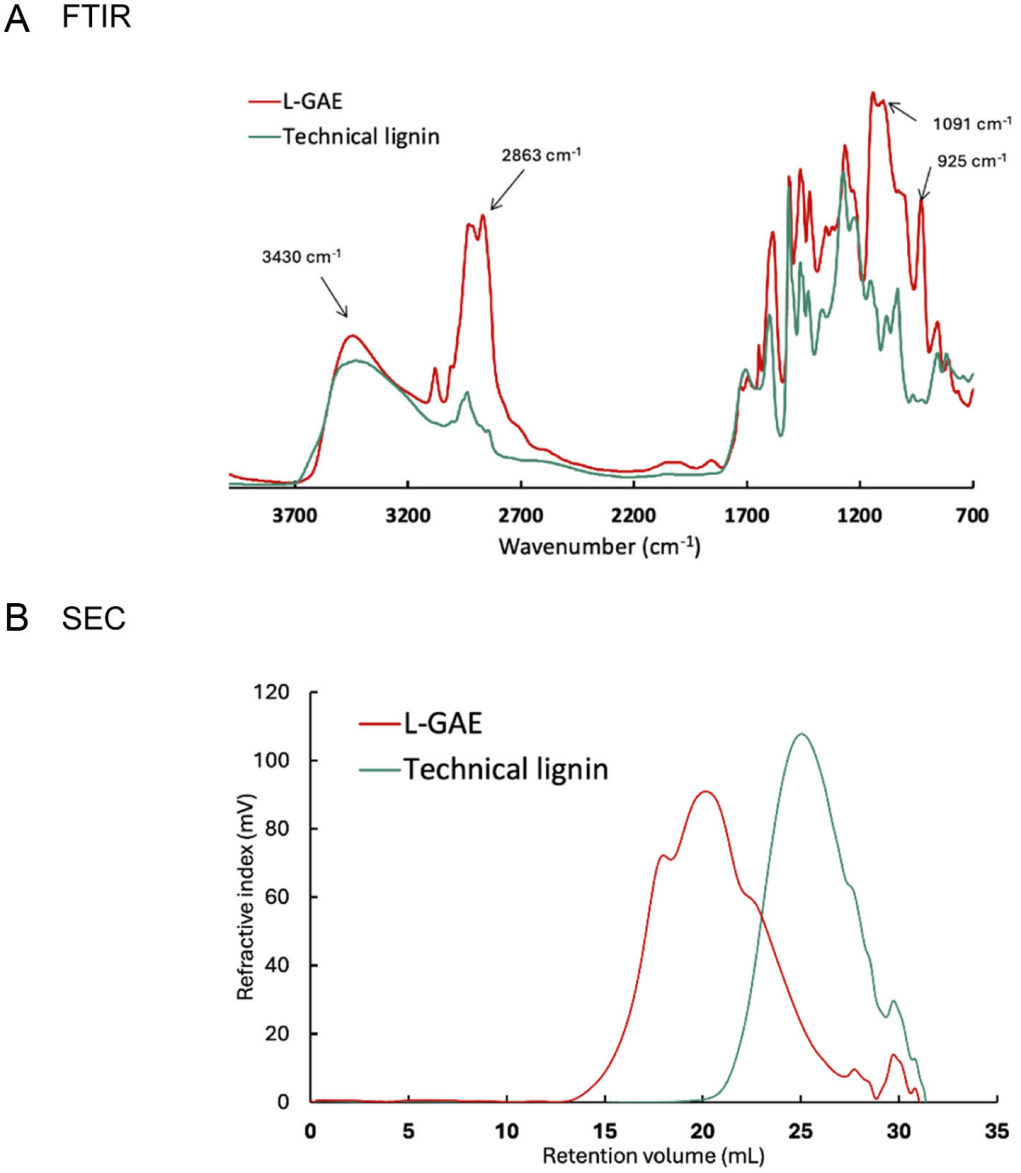
Analytical results on the sample in Table 3, entry 2: (A) Comparison of FTIR spectra of technical Kraft lignin (green) and modified Kraft lignin L‐GAE (red). (B) Comparison of SEC spectra of technical (green) and modified Kraft lignin L‐GAE (red).

Following these very promising results for the functionalization of lignin using GAE, an in‐depth analytical study was carried out on L‐GAE isolated from the protocol in entry 2 of Table 3. The FTIR spectrum of L‐GAE logically shows the absence of a band at 1790 cm^−1^ corresponding to the carbonyl groups compared with L‐AGC (Figure 2A). In contrast, the bands at 2900 and 1100 cm^−1^ corresponding to alkane chains coupled to ethers are very strong and validate the presence of glycerol moieties included in the material. In addition, the strong band at 925 cm^−1^ also confirms the high proportion of unsaturation. The grafting efficiency has an impact on SEC measurements (Figure 2B) with a significant increase in *M*_n_ to 6800 g/mol, much higher than the starting Kraft lignin (650 g/mol). These masses are also superior to L‐AGC lignin (4200 g/mol), in line with the higher levels of unsaturation obtained with epoxide ring opening. However, the polydispersity index is higher on the L‐GAE (*Đ *= 6.3) than on the L‐AGC (*Đ *= 2.6), suggesting that overopenings have a stronger impact on dispersity than transcarbonations. Note that additional characterizations by ^13^C and HSQC NMR have also confirmed the structure and are available in Supporting Information (Figures S19 and S20).

**FIGURE 3:**
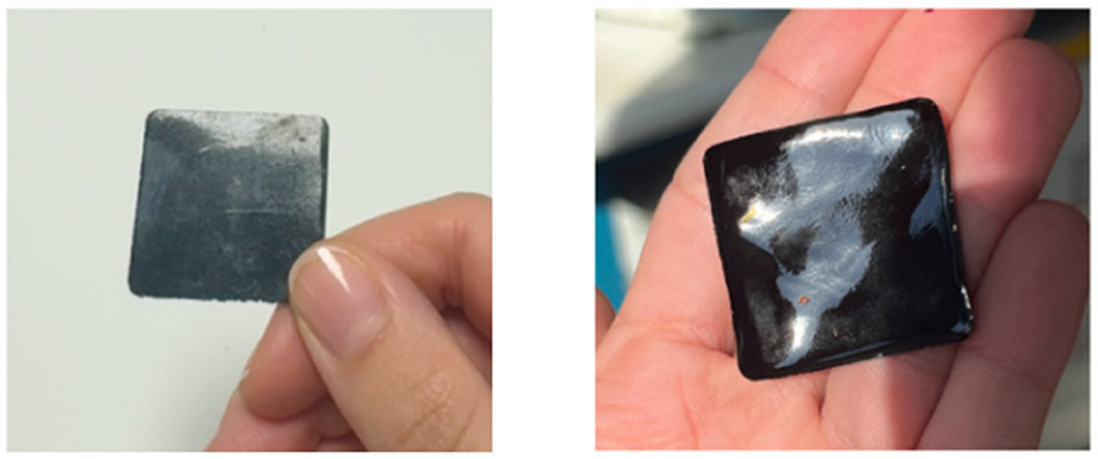
L‐GAE (left) and L‐GTE (right) mold at 130 °C during 6 h.

Interestingly, the thermal properties of L‐GAE are strongly affected by the proportion of added chains. The glass‐transition temperature for L‐GAE drops to 51 °C, well below L‐AGC (94 °C) or technical Kraft lignin (155 °C). Although the degree of substitution of hydroxyl groups remains similar, the absence of a cross‐linking node (transcarbonation) allows L‐GAE lignin to be shaped under the action of heat, being particularly viscous above 120 °C (Figure 3).

**FIGURE 4:**
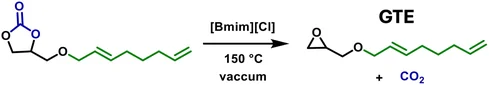
Synthesis of GTE from the telomerization product of 1,3‐butadiene on glycerol carbonate.

The robustness of this grafting method was evaluated on different lignin sources (OH quantification in Table S1). Three types of industrially available lignin were selected (Kraft, Organosolv, and Soda). The grafting protocol optimized with GAE was applied to these commercially available lignins, and the same analytical study was carried out using ^1^H and ^31^P NMR as well as SEC and DSC (Table 4). Another reactant with a longer carbon chain was also synthesized from GC telomerized with 1,3‐butadiene based on our previous work [44]. This compound was then decarboxylated following the same method as used to obtain GAE. Vacuum was in that particular case used to distill glycidyl telomer ether (GTE), which has a higher boiling point than GAE (Figure 4).

**TABLE 4:** Scope of GAE and GTE grating on lignins from different extraction processes.

			Substitution degree^a^						SEC		DSC
	Entry	Lignin	OH tot.	OH Al.	OH Ph.	COOH	Lignin wt%	Grafted chains per OH	*M*_n_ b, g/mol	*Đ* b	*T*_g_, °C^c^
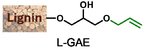	1	Kraft	90	55	100	100	47	1.4	6800	3.5	50
	2	Organosolv	82	47	100	100	52	1.2	5300	3.1	60
	3	Soda	81	17	100	100	56	1.1	6200	3.7	62
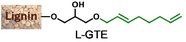	4	Kraft	76	2	99	100	53	0.7	6100	4.2	54
	5	Organosolv	66	<1	100	100	47	0.9	4800	2.4	50
	6	Soda	78	5	100	100	49	0.9	6500	3.7	55

*Note* *:* Conditions: 0.250 g of lignin, 3.5 eq. of the respective reactant; 0.05 eq of K_2_CO_3_, 0.5 mL of dioxane; Schlenk equipped with a Rotaflow stopcock.

a Determined by ^31^P NMR.

b Determined by SEC.

c Determined by DSC.

The scope was performed according to the protocol defined in Table 3 entry 2, i.e., 250 mg of lignin mixed with 3.5 equivalents of epoxide reagent and 0.05 eq. of K_2_CO_3_ in 0.5 mL of dioxane for 3 h. The substitution degrees measured on the functionalized Organosolv lignin with GAE are similar to the one of the Kraft lignin, with 82% of total OH groups converted, including 47% of aliphatic hydroxyl groups (Table 4, entry 2). The overopening was also observed, reaching 1.2 chains per OH group. GAE grafting is also effective on Soda lignin, with 81% substitution of total OH despite a lower conversion of aliphatic hydroxyls (17%) which are less abundant initially in this type of lignin. The various reactions performed using GTE with longer chains showed a slight drop in overall efficiency with substitution degrees of total OH of 76%, 66%, and 78%, respectively, for Kraft, Organosolv, and Soda. This is mainly due to the lack of reactivity of aliphatic hydroxyl groups in the case of GTE. This could be explained by the lower availability of aliphatic groups due to the steric hindrance of the added chains. On the other hand, the advantage is that epoxide group overopening reactions are limited, with no more than 0.9 chains per OH group, close to the chains added to lignin OH groups. These results confirm the versatility of this methodology across different types of lignin and with different chain sizes, bearing one or two available reactive unsaturations.

Thermal analysis by DSC shows similar glass‐transition temperatures (*T*_g_) between all the modified lignins. These *T*_g_ are ranging from 50 to 62 °C. From a thermal point of view, the lower OH substitution of L‐GTE is compensated by a higher chain size. As a result, each modified lignin contains around 50% by mass of grafted chains (see Lignin wt%). Lignins modified following these protocols have a significantly reduced viscosity when heated. This allows them to be easily formulated without solvents. Moreover, their unsaturation can be exploited to develop interesting biobased materials. Furthermore, differences in reactivity and their consequences on properties are also highlighted by SEC analyses. The average molecular weights (*M*_n_) of L‐GAE lignins (between 5300 and 6800 g/mol) and those of L‐GTE lignins (between 4800 and 6500 g/mol) are close. Thus, L‐GAE lignins have molecular weights similar to those of L‐GTE lignins, despite having shorter allylic chains, which can be explained by the presence of epoxide overopenings. In all cases, the dispersity *Đ* of the lignins remains in the range of 2.4–4.2. It should be noted that the Mn values and dispersity are slightly lower for Organosolv lignins, which can be explained by the lower average molar mass of the technical organosolv lignin (620 g/mol) compared to that of Kraft (960 g/mol) and Soda (730 g/mol) lignins.

**FIGURE 5:**
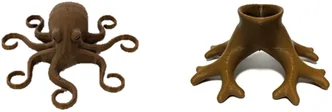
3D printed objects with PA12 and 10% of modified lignin (left GAE–right GTE).

The added chains provide lignin with improved lipophilicity and represent a good opportunity to improve compatibility in polymer matrices. A proof of concept, to provide an initial insight into the behavior of modified lignins in a practical application, has been obtained by formulating these modified lignins in a blend with polyamide 12 (PA12) in a twin‐screw extruder to obtain 3D printing filaments. Modified lignins have been shown to be suitable for extruder formulation, in contrast to technical lignins, which tend to emit smoke at relatively low weight loading. TGA confirms that both modified lignins exhibit minimal mass loss at 220 °C (≤2%, vs. 6% for unmodified Kraft), demonstrating their thermal inertness during melt processing. This absence of early volatile release greatly limits smoke formation. Therefore, blend formulations were pelletized and extruded again at 220 °C to achieve a constant diameter of 1.75 mm, which is the most common diameter for feedstock materials used in 3D printing. Filaments of PA12 with 10% of L‐GAE and 10% of L‐GTE were printed. The displayed objects illustrate the appropriate behavior of the materials during printing (Figure 5). Polyamides are typically difficult to 3D print due to their high melting temperature and rapid shrinkage. The successful printing trail highlights suggest that the addition of modified lignin improves printability, surface finish, and adhesion to the print bed due to the thermal behavior of modified lignins in the blend.

Thus, in addition to adding biobased material components to materials, lignin also provides a performance gain for this kind of application. An in‐depth study of the mechanical properties of different formulations, combined with a 3D printing study, is currently in progress.

## 4 Conclusion

New strategies for lignin functionalization have been developed with ambitions of adding a large amount of unsaturation, which is useful for a wide range of biobased applications. Allylation of GCT was optimized with a ruthenium catalyst (RuCp(PPh_3_)_2_Cl) to produce ready‐to‐graft molecules. This catalysis, without salts or coproducts, showed a good efficiency of 78% yield under mild conditions and ultimately without the need of silver salts. This molecule, derived from glycerol, was then grafted onto lignins and model molecules by a ring‐opening / decarboxylation reaction that involves the cyclic carbonate and hydroxyl groups. Although this reaction proved to be effective with the conversion of all phenols in Kraft lignin, it inevitably led to cross‐linking nodes via undesirable transcarbonation reactions. To avoid this drawback, cyclic carbonates have been converted into epoxides. This conversion was achieved using a straightforward imidazolium salt‐catalyzed method with good yields. Two unsaturated glycidyl ethers were grafted on Kraft lignin with substitution degrees of up to 90% of the total hydroxyl groups. Under these conditions, no cross‐linking was observed. Epoxides also reacted with aliphatic hydroxyl groups, leading to moderate overopening. This side reaction could be limited by appropriate adjustment of the reaction parameters. Furthermore, this methodology has also been successfully applied to Organosolv and Soda lignins, demonstrating the feasibility of this strategy for most commercial lignins. These lignins have interesting thermal properties, allowing them to be shaped under the effect of temperature. The abundance of grafted unsaturations in lignin opens up a wide range of applications in the field of materials. The proof‐of‐concept suggest that these lignins could be incorporated into polymer blends with PA12 for additive manufacturing. These blends are currently undergoing further studies.

## Funding

This study was supported by the Agence Nationale de la Recherche (ANR‐22‐CE10‐0010).

## Conflicts of Interest

The authors declare no conflicts of interest.

## Supporting information

Supplementary Material

## Data Availability

The data that supports the findings of this study are available in the supporting information of this article.
