# Supplementary material for: Highly Unsaturated Lignin Through Clean and Efficient Ru‐Catalyzed Glycerol Carbonate Allylation and Ring‐Opening Reaction Strategies
Source: ChemSusChem. 2026 Sep 22;19(18):e71047. doi: 10.1002/cssc.71047 (PMC13598274; doi:10.1002/cssc.71047)
Supplement: Supplementary file 1 [file CSSC-19-e71047-s001.pdf]

Highly unsaturated lignin through clean and efficient Ru-catalysed glycerol carbonate allylation and ring-opening reaction strategies.

Claudie Simon,*[a] Tiphaine Richard,[a,b] Sofiane Guessasma,[c] Mathieu Sauthier,[a] Klara Jastak[b] and Clément Dumont*[a,b]

Supporting information

Contents

1. Allylation of glycerol carbonate ... 3

Figure ESI 1 - ^{1}H NMR of allyl glycerol carbonate (GCA) ... 3
Figure ESI 2 - ^{13}C NMR of allyl glycerol carbonate (GCA) ... 4
Figure ESI 3 – ^{1}H NMR of crude after evaporation ... 4

2. Characterization of the native lignins ... 5

Figure ESI 4 - DSC of technical lignin (Kraft) ... 5
Figure ESI 5 - ^{31}P NMR of Kraft lignin ... 6
Figure ESI 6 - ^{31}P NMR of Soda lignin ... 6
Figure ESI 7 - ^{31}P NMR of Organosolv lignin ... 7

3. Functionalisation of lignin with allyl glycerol carbonate ... 8

3.1. Characterisation of L-AGC ... 8

Figure ESI 8 - ^{13}C NMR of allyl glycerol carbonate grafted on kraft lignin ... 8
Figure ESI 9 - HSQC of allyl glycerol carbonate grafted on kraft lignin ... 9
Figure ESI 10 - ^{31}P NMR of allyl glycerol carbonate grafted on kraft lignin ... 9
Figure ESI 11 - DSC of allyl glycerol carbonate grafted on kraft lignin (endo down) ... 10
Figure ESI 12 - TGA of allyl glycerol carbonate grafted on kraft lignin ... 10

3.2. Calculation of substitution degrees ... 10

Figure ESI 13 – Line fitting of the hydroxyl group of modified lignin ... 11

4. Decarboxylation of allyl glycerol carbonate into glycidyl allyl ether ... 12

Figure ESI 14 - ^{1}H NMR of glycidyl allyl ether (GAE) ... 12
Figure ESI 15 - ^{13}C NMR of glycidyl allyl ether (GAE) ... 13
Figure ESI 16 – Decarboxylation reaction setup ... 13

5. Study on Guaiacol ... 13

Figure ESI 17 - ^{1}H NMR of glycidyl allyl ether grafted on guaiacol ... 14
Figure ESI 18 - ^{13}C NMR of glycidyl allyl ether grafted on guaiacol ... 15

6. Functionalisation of lignin with glycidyl allyl ether ... 15

Figure ESI 19 - ^{13}C NMR of glycidyl allyl ether grafted on kraft lignin ... 15
Figure ESI 20 - HSQC of glycidyl allyl ether grafted on kraft lignin ... 16
Figure ESI 21 - ^{31}P NMR of glycidyl allyl ether grafted on kraft lignin ... 16
Figure ESI 22 - DSC of glycidyl allyl ether grafted on kraft lignin (endo down) ... 17
Figure ESI 23 - TGA of glycidyl allyl ether grafted on kraft lignin ... 17
Figure ESI 24 - ^{1}H NMR of GAE after reaction without lignin ... 18

Figure ESI 25 - Comparison of ^{1}H NMR of L-GAE with 1.6 grated chains (red) and L-GAE with 0.6 grafted chains (green) ... 18

7. Functionalisation with different types of lignin with GAE ... 19

Figure ESI 26 - ^{1}H NMR of GAE grafted on Organosolv lignin ... 19
Figure ESI 27 - ^{31}P NMR of GAE grafted on Organosolv lignin ... 19
Figure ESI 28 - ^{1}H NMR spectra of GAE grafted on Soda lignin ... 20
Figure ESI 29 - ^{31}P NMR spectra of GAE grafted on Soda lignin ... 20

8. Functionalisation of lignin with GTE ... 21

Figure ESI 30 : ^{1}H NMR of GTE grafted on Kraft lignin ... 21
Figure ESI 31 - ^{31}P NMR of GTE grafted on Kraft lignin ... 21
Figure ESI 32 : ^{1}H NMR of GTE grafted on Organosolv lignin ... 22
Figure ESI 33 - ^{31}P NMR of GTE grafted on Organosolv lignin ... 22
Figure ESI 34 : ^{1}H NMR of GET grafted on Soda lignin ... 23
Figure ESI 35 - ^{31}P NMR of GTE grafted on Soda lignin ... 23

9. ATG comparison of Kraft and modified lignins ... 24

Figure ESI 36 – TGA of technical Kraft Lignin ... 24
Figure ESI 37 – TGA of GAE grafted on Kraft Lignin ... 24
Figure ESI 38 – TGA of GTE grafted on Kraft Lignin ... 24

1. Allylation of glycerol carbonate

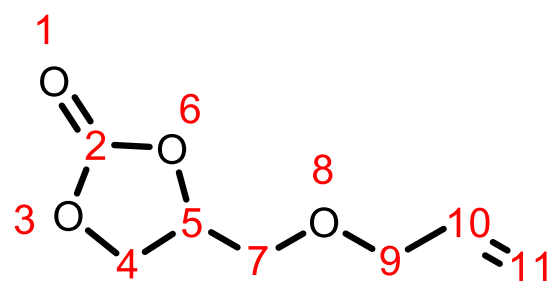

^{1}H NMR (300 MHz, Chloroform-*d*) δ 5.87 (ddt, *J* = 17.3, 10.3, 5.7 Hz, 1H) [10], 5.36 – 5.18 (m, 2H) [11], 4.82 (ddt, *J* = 8.1, 6.1, 3.9 Hz, 1H) [5], 4.55 – 4.36 (m, 2H) [4], 4.06 (dq, *J* = 5.7, 1.5 Hz, 2H) [9], 3.66 (qd, *J* = 10.9, 3.9 Hz, 2H) [7].

^{13}C NMR (75 MHz, $CDCl_3$) δ 155.03 [2], 133.76 [10], 118.18 [11], 75.08 [5], 72.78 [9], 68.96 [7], 66.43 [4].

HRMS m/z: Calculated for $[M+H]^+$ $C_7H_{10}O_4$ 159.0652; Found 159.0649 (Δm = 0.0003 m/z)

Isolated product obtained by column chromatography on silica gel with petroleum ether/ethyl acetate 80 : 20 eluant – 75% isolated yield

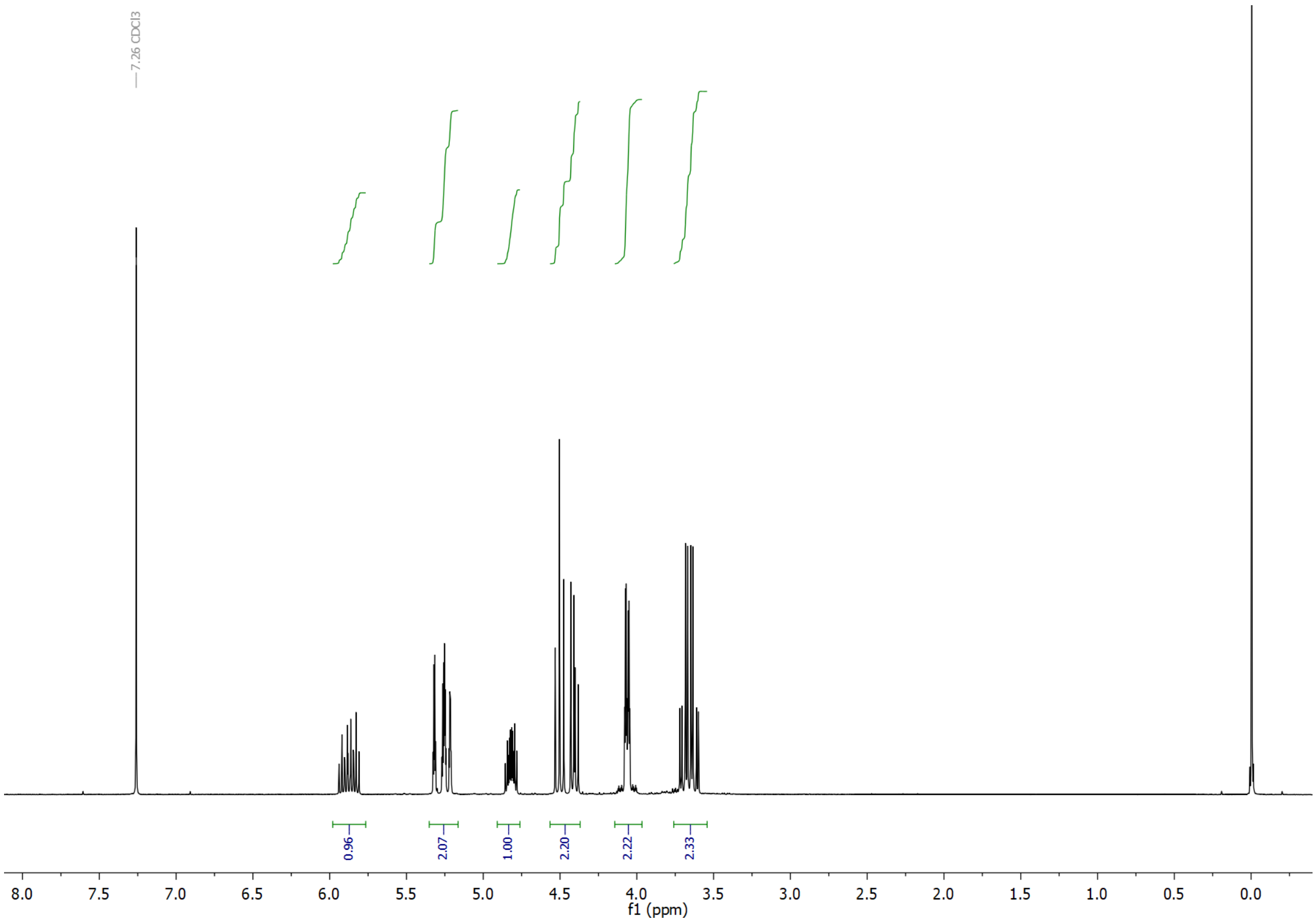

Figure ESI 1 - ^{1}H NMR of allyl glycerol carbonate (GCA)

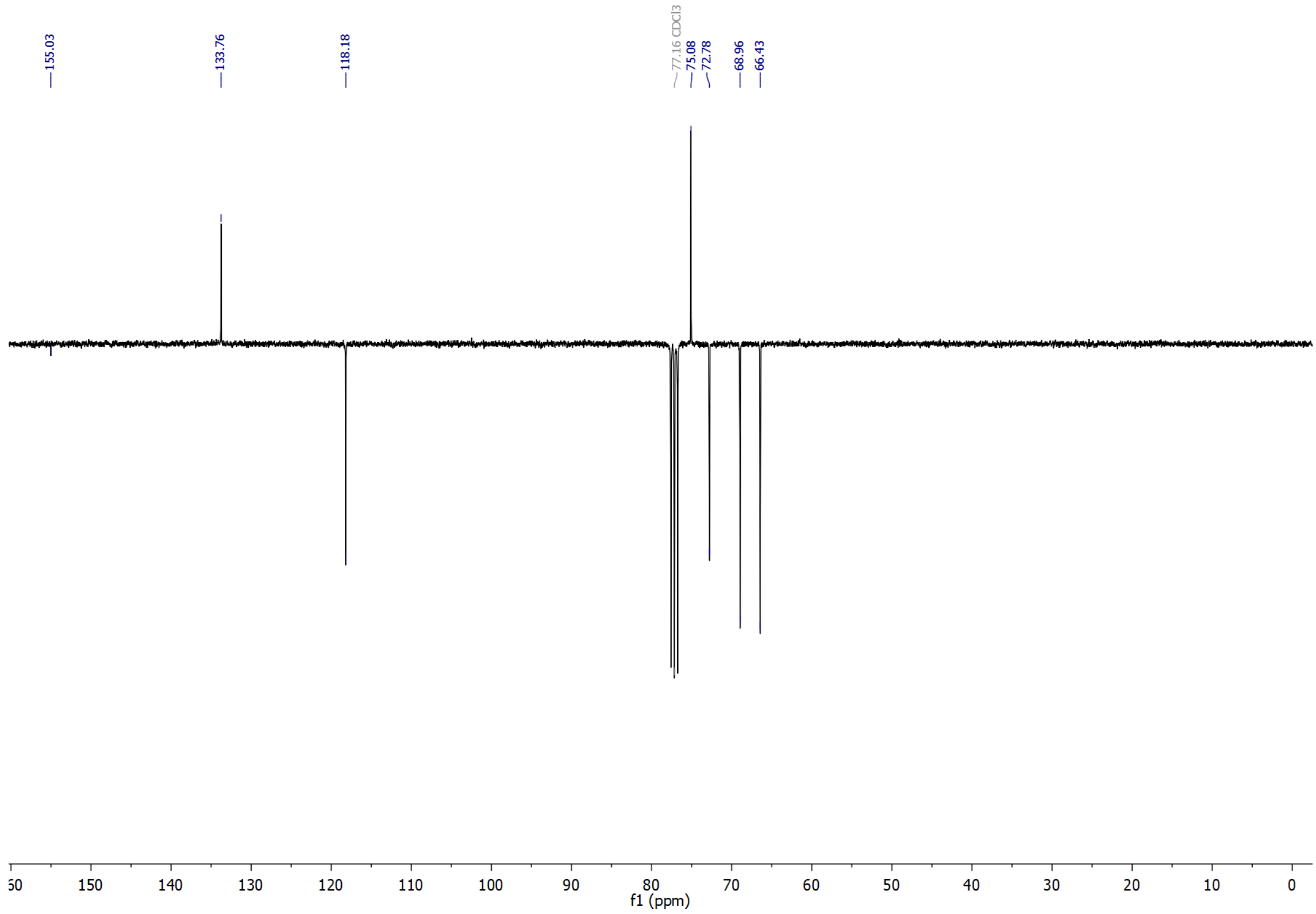

Figure ESI 2 - ^{13}C NMR of allyl glycerol carbonate (GCA)

Crude at the end of reaction is evaporated to remove solvent (THF), then is analysed in ^{1}H NMR to determine yield. NMR ^{1}H was realised in DMSO-D$_6$. In this solvent, proton 3 of glycerol carbonate and allyl glycerol carbonate does not have the same chemical displacement.

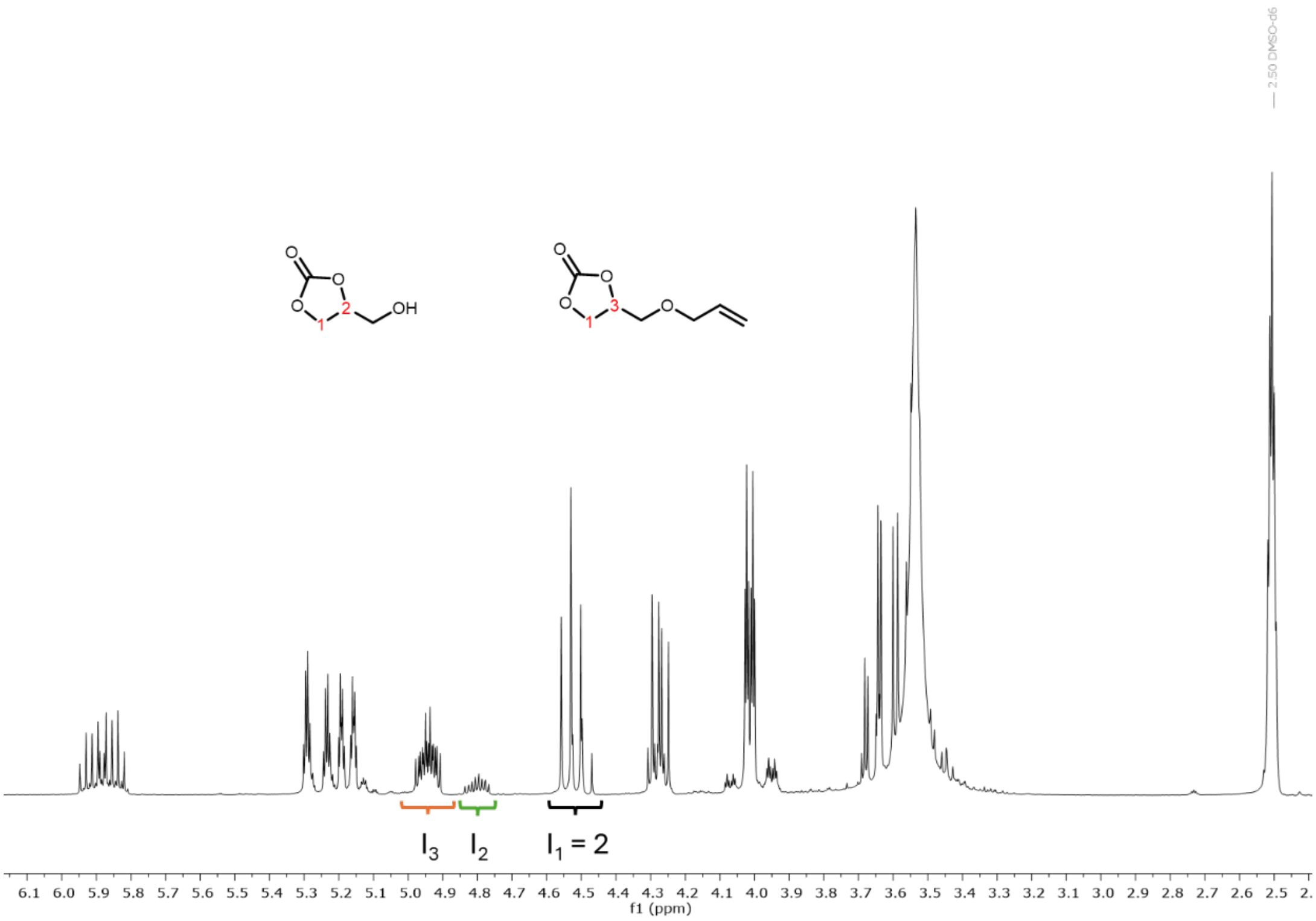

Figure ESI 3 – ^{1}H NMR of crude after evaporation

The signal from proton 1 is fixed at 2 because it corresponds to 2 protons. This signal is used as a reference peak.

If 100% of the glycerol carbonate is converted, the integration of proton 3 is equal to 1. The yield is calculated on this assumption

$$\text{Yield GCA (\%)} = \frac{I_3}{2} \times 100$$

$$\text{Conversion GCA (\%)} = \frac{(1-I_2)}{2} \times 100$$

2. Characterization of the native lignins

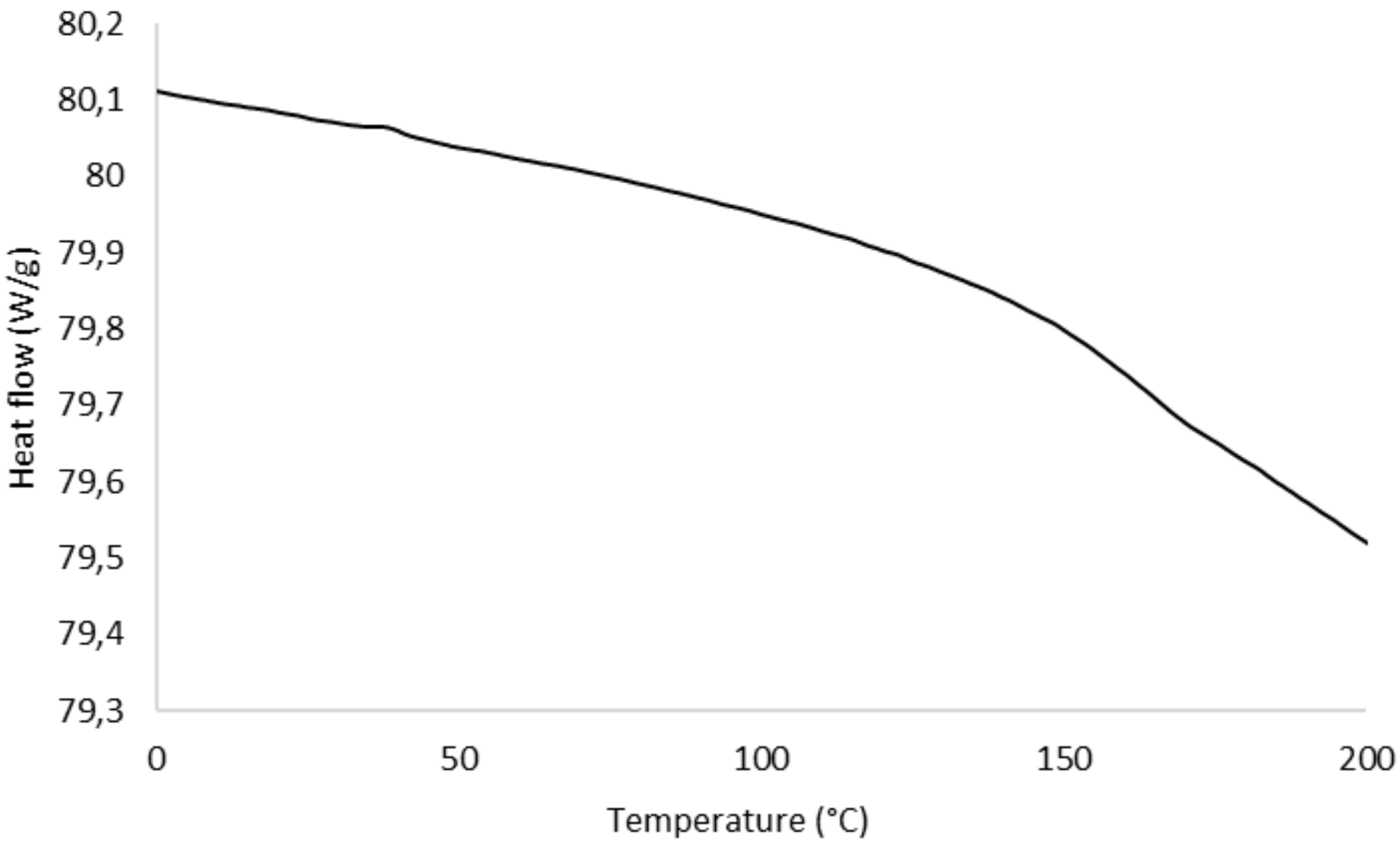

Figure ESI 4 - DSC of technical lignin (Kraft)

^{31}P NMR spectra of Kraft, Organosolv and Soda lignin

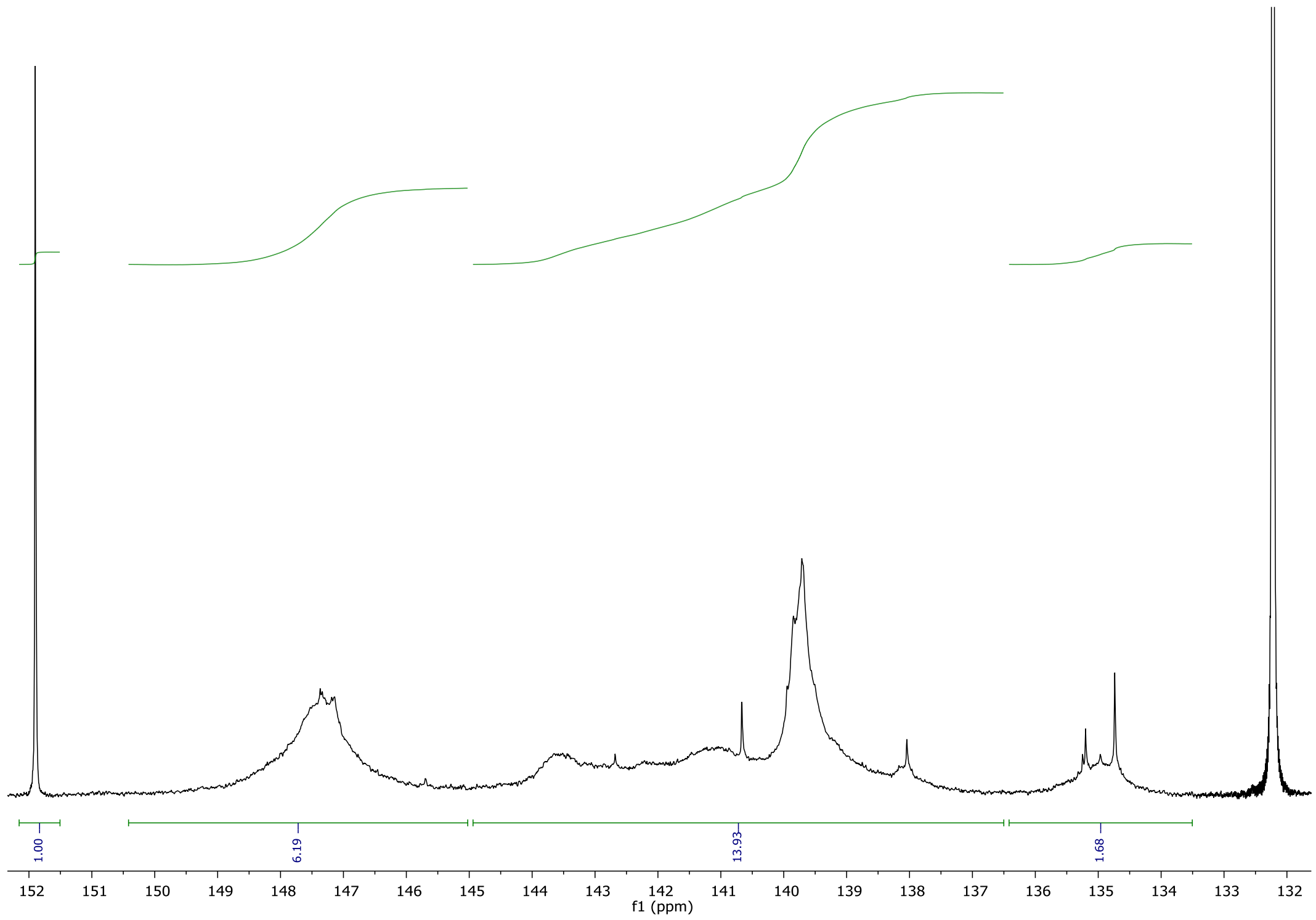

Figure ESI 5 - ^{31}P NMR of Kraft lignin

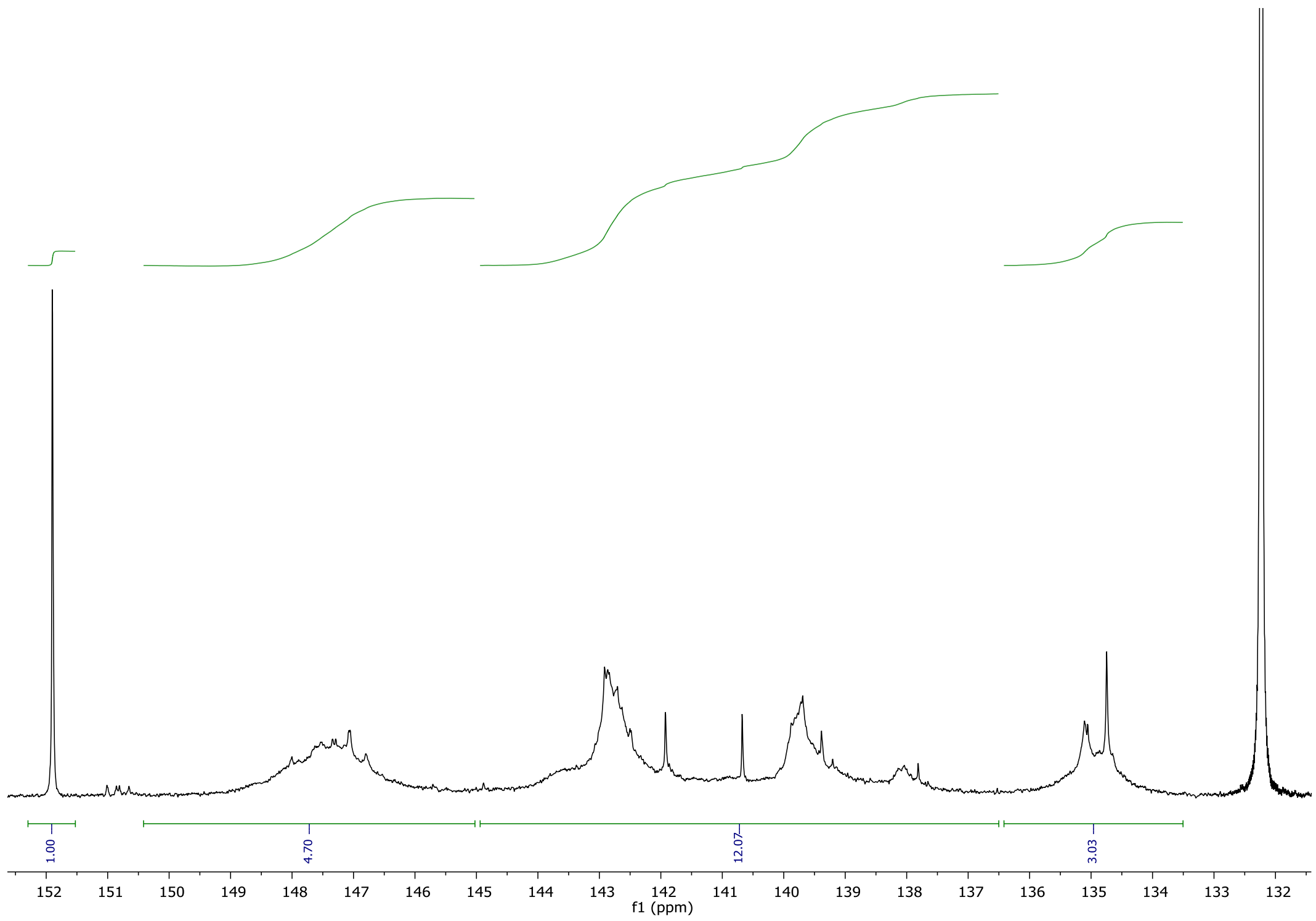

Figure ESI 6 - ^{31}P NMR of Soda lignin

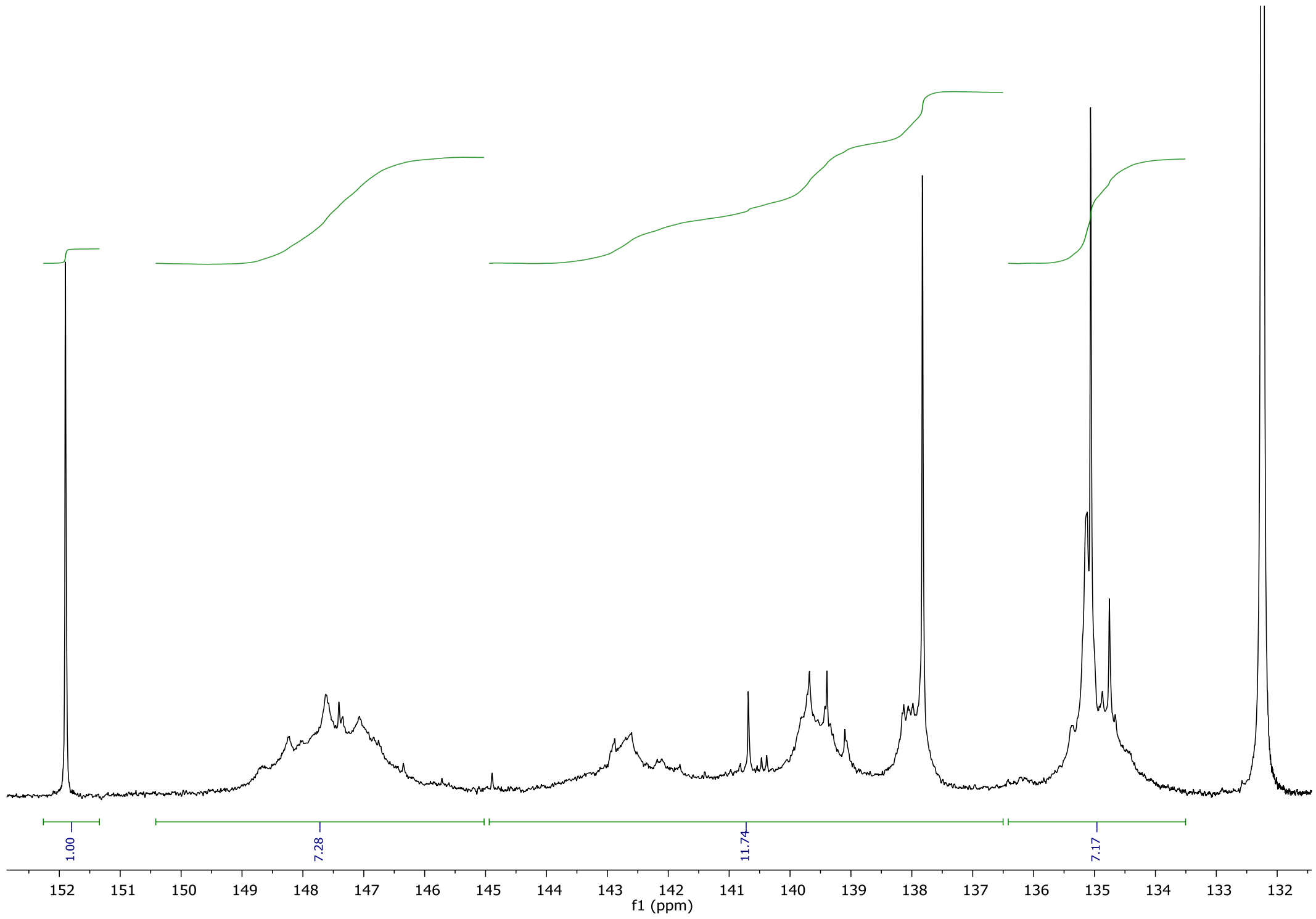

Figure ESI 7 - ^{31}P NMR of Organosolv lignin

Table ESI 1 - 31P analysis of lignins - titration of hydroxyl groups

Lignin	OH aliphatic mmol/g	Phenolic OH mmol/g	Total OH mmol/g	Carboxylic acid mmol/g
Kraft	1,9	4,2	6,1	0,5
Soda	1,5	3,9	5,4	1,0
Organosolv	2,4	3,1	5,5	1,5

3. Functionalisation of lignin with allyl glycerol carbonate

3.1. Characterisation of L-AGC

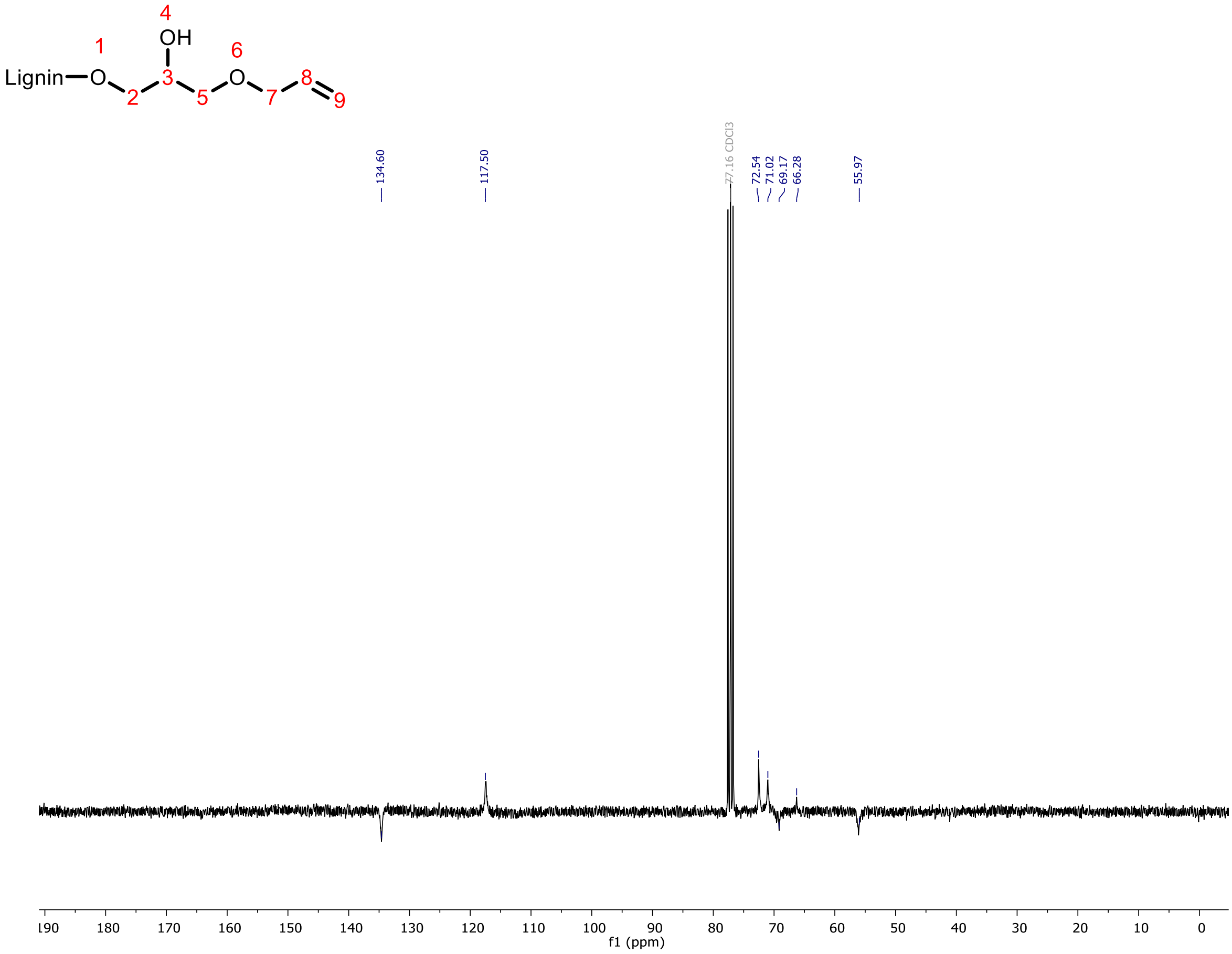

Figure ESI 8 - ^{13}C NMR of allyl glycerol carbonate grafted on kraft lignin

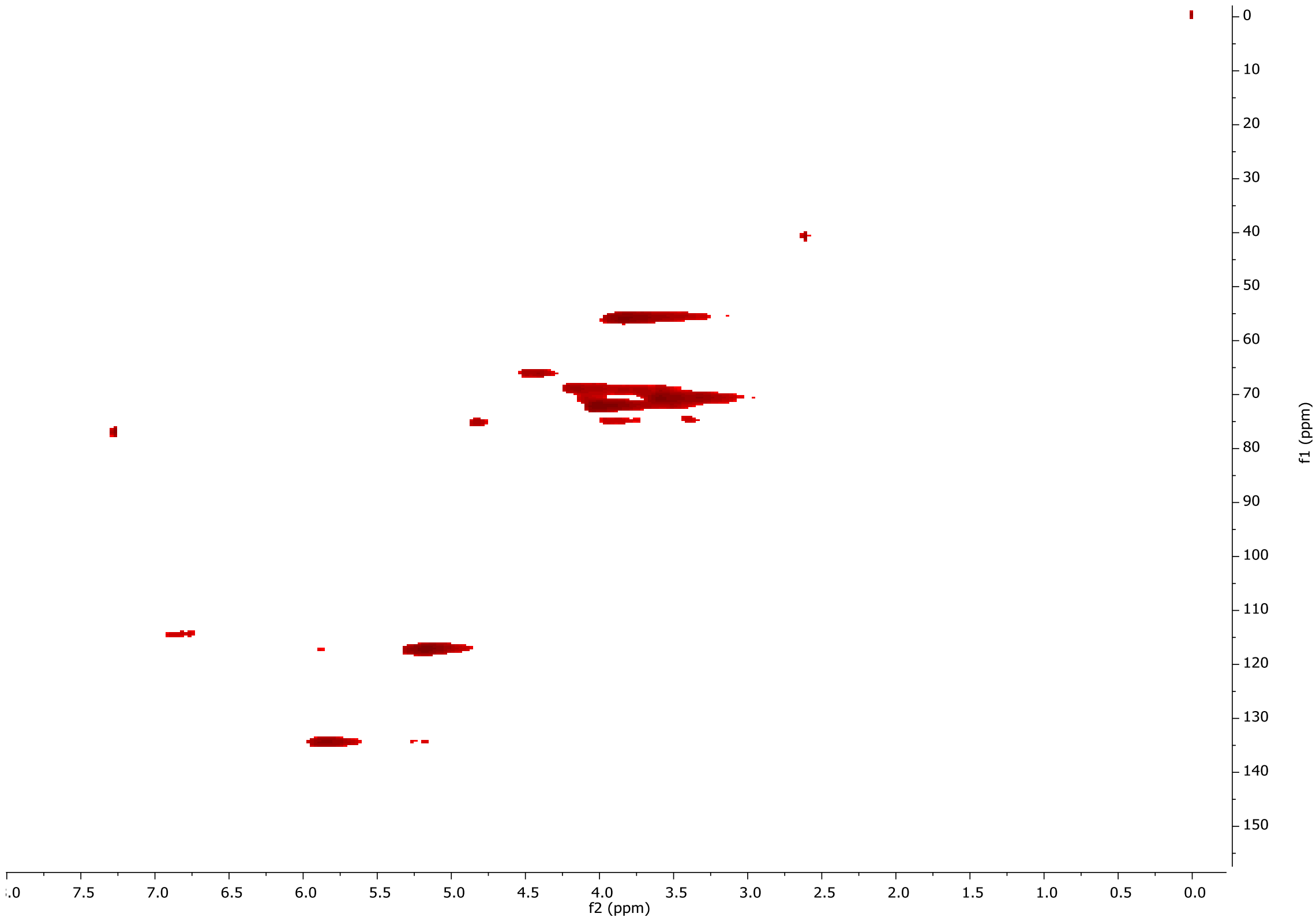

Figure ESI 9 - HSQC of allyl glycerol carbonate grafted on kraft lignin

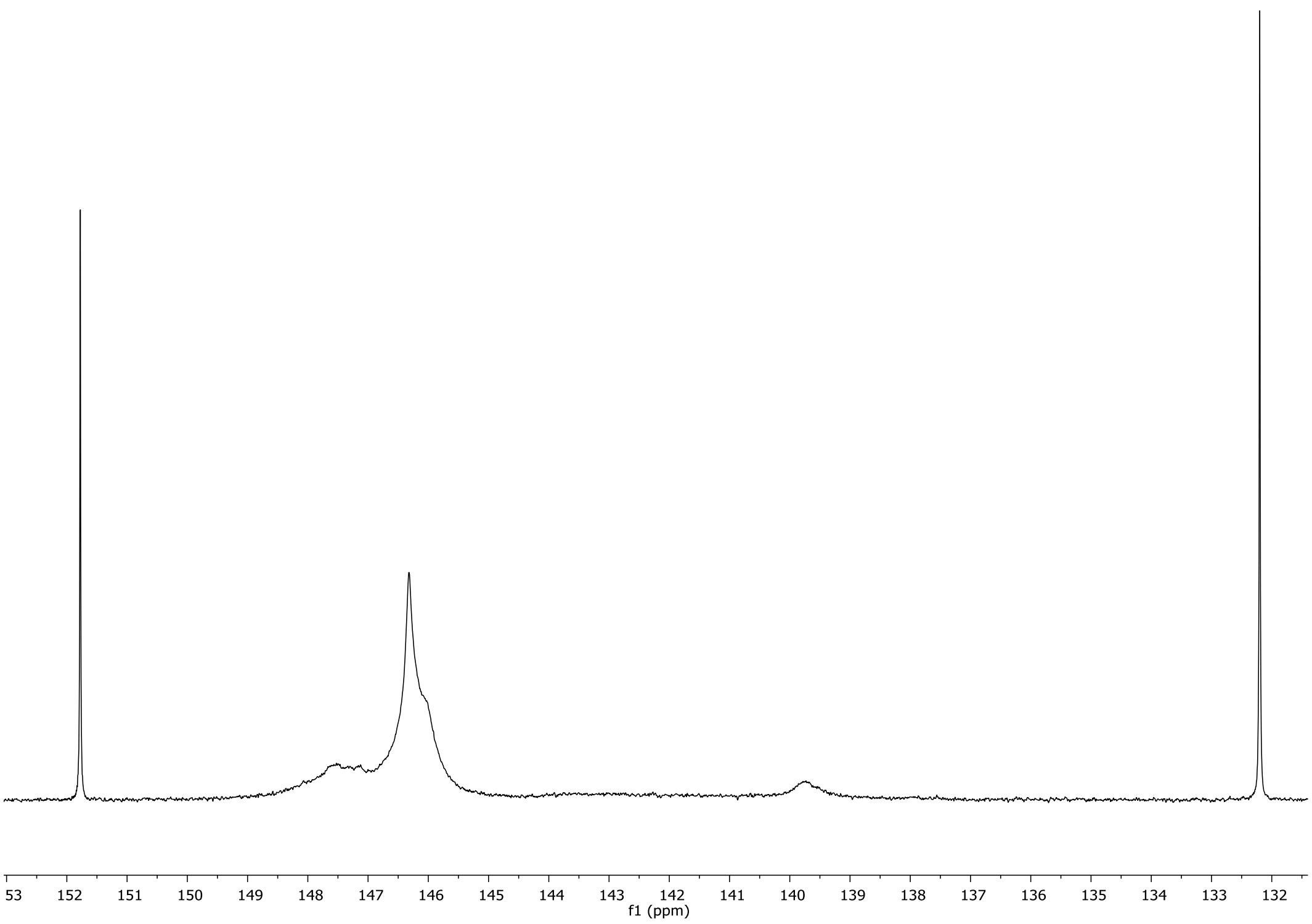

Figure ESI 10 - ^{31}P NMR of allyl glycerol carbonate grafted on kraft lignin

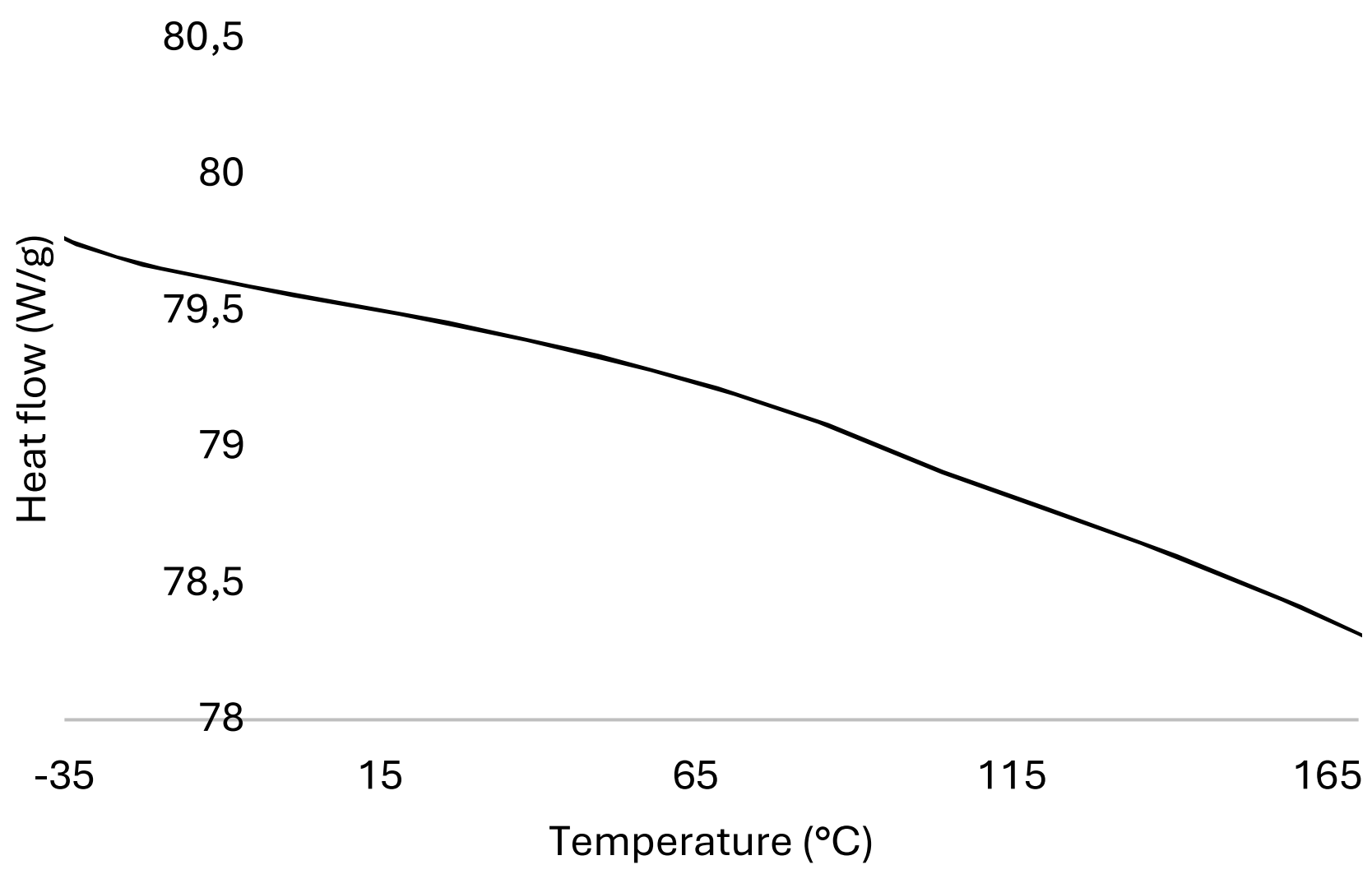

Figure ESI 11 - DSC of allyl glycerol carbonate grafted on kraft lignin (endo down)

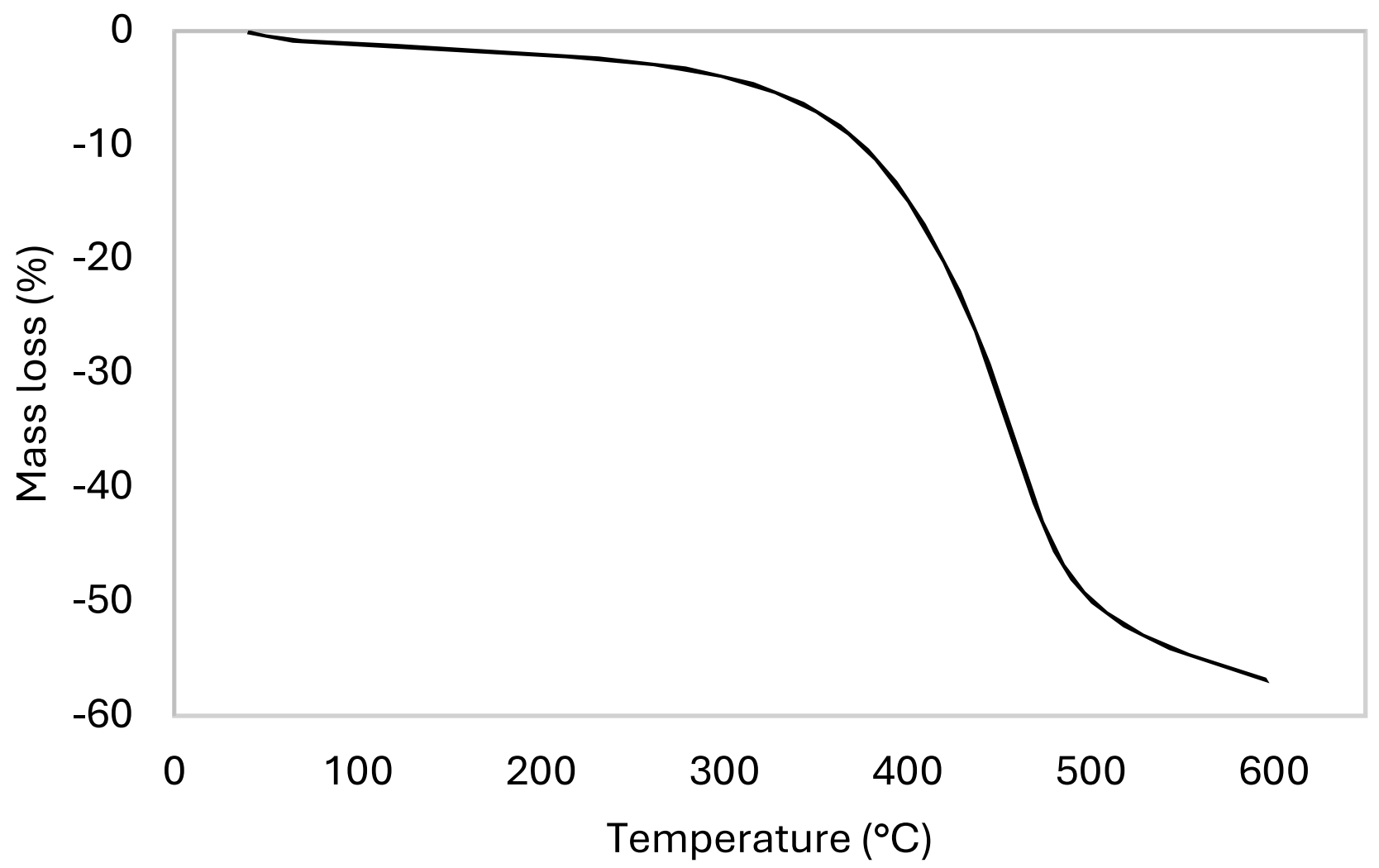

Figure ESI 12 - TGA of allyl glycerol carbonate grafted on kraft lignin

3.2. Calculation of substitution degrees

^{31}P NMR analysis is used to determine the amount of OH in the sample by transforming OH into a phosphorus-containing group. An internal standard of known quantity (1.10^{-5} moles) is added to lignin (approx. 30 mg) in a hemolysis tube. Substitution of carboxylic acid group was complete in all samples.

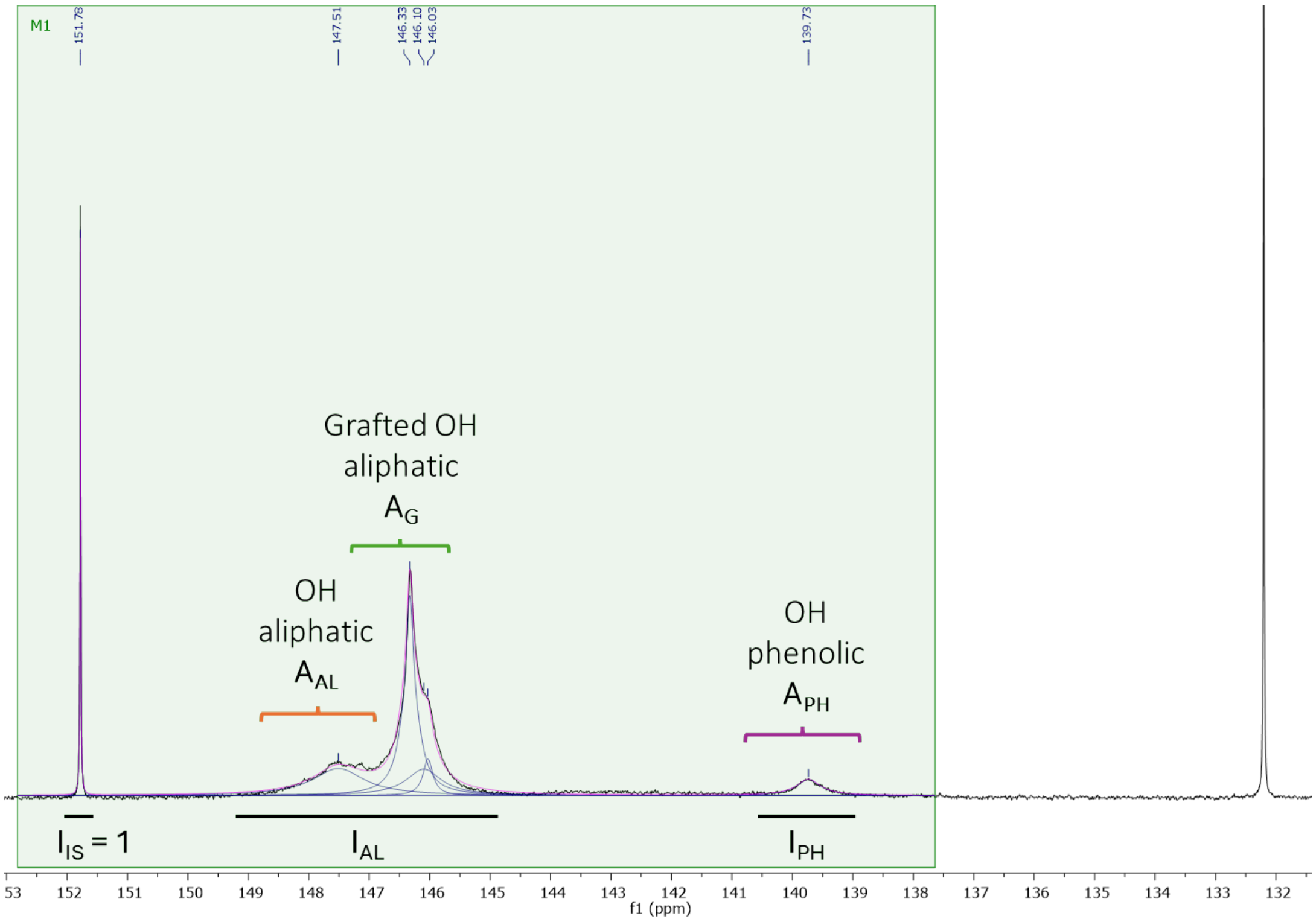

Figure ESI 13 – Line fitting of the hydroxyl group of modified lignin

A_{PH} = Peak area of the unmodified phenolic hydroxyl groups

A_{AL} = Peak area of the unmodified aliphatic hydroxyl groups

A_G = Peak area of the grafted aliphatic hydroxyl groups

I_{IS} = ^{31}P NMR integration of internal standard. Fixed at 1.

I_{PH} = ^{31}P NMR integration of unmodified phenolic hydroxyl groups as shown in Figure ESI 10 relative to the internal standard

I_{AL} = ^{31}P NMR integration of total aliphatic hydroxyl groups as shown in Figure ESI 10 relative to the internal standard

Substitution degree of OH phenolics

$$\%\text{Substitution of OH ph.} = \frac{1-\left(\frac{A_{PH}}{A_G+A_{AL}}\right)\times 100}{72}\times 100$$

72% represent the proportion of OH phenolics in technical lignin before functionalisation.

Substitution degree of OH aliphatics

$$\%\text{Substitution of OH al} = \frac{1-\left(\frac{A_{AL}}{A_G+A_{AL}}\right)\times 100}{24}\times 100$$

24% represent the proportion of OH aliphatic in technical lignin before functionalisation.

Total substitution degree

$$\%\text{Substitution total} = \frac{(\%\text{substitution OH al}\times\text{initial OH al})+(\%\text{substitution OH ph}\times\text{initial OH ph})+(\%\text{substitution COOH}\times\text{initial COOH})}{\text{Total OH initial}}$$

Quantification of OH in modified lignin (mmol/g):

$$OH_{\text{modified lignin}}(\text{mmol/g}) = \frac{(I_{AL}+I_{PH})\times 10^{-5}}{\text{sample weight}}\times 1000$$

Calculation of native lignin in the sample (%):

$$\%\text{lignin} = \frac{OH_{\text{modified lignin}}}{OH_{\text{native lignin}}}\times 100$$

Calculation of the grafted chains per OH:

$$\text{Grafted chains per OH} = \frac{\left(\frac{1-\%\text{lignin}}{\text{Molecular mass of added chains}}\right)}{OH_{\text{modified lignin}}}$$

4. Decarboxylation of allyl glycerol carbonate into glycidyl allyl ether

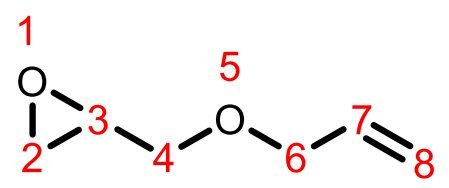

^{1}H NMR (300 MHz, Chloroform-*d*) δ 5.91 (ddt, *J* = 17.3, 10.4, 5.6 Hz, 1H) [7], 5.29 (dq, *J* = 17.2, 1.7 Hz, 1H) [8], 5.19 (dq, *J* = 10.4, 1.4 Hz, 1H) [8], 4.14 – 3.93 (m, 2H) [6], 3.72 (dd, *J* = 11.4, 3.1 Hz, 1H) [4], 3.41 (dd, *J* = 11.4, 5.8 Hz, 1H) [4], 3.16 (ddt, *J* = 5.8, 4.2, 2.9 Hz, 1H) [3], 2.80 (dd, *J* = 5.0, 4.2 Hz, 1H) [2], 2.61 (dd, *J* = 5.0, 2.7 Hz, 1H) [2].

^{13}C NMR (75 MHz, $CDCl_3$) δ 134.40 [7], 117.35 [8], 72.27 [6], 70.78 [4], 50.79 [3], 44.34 [2].

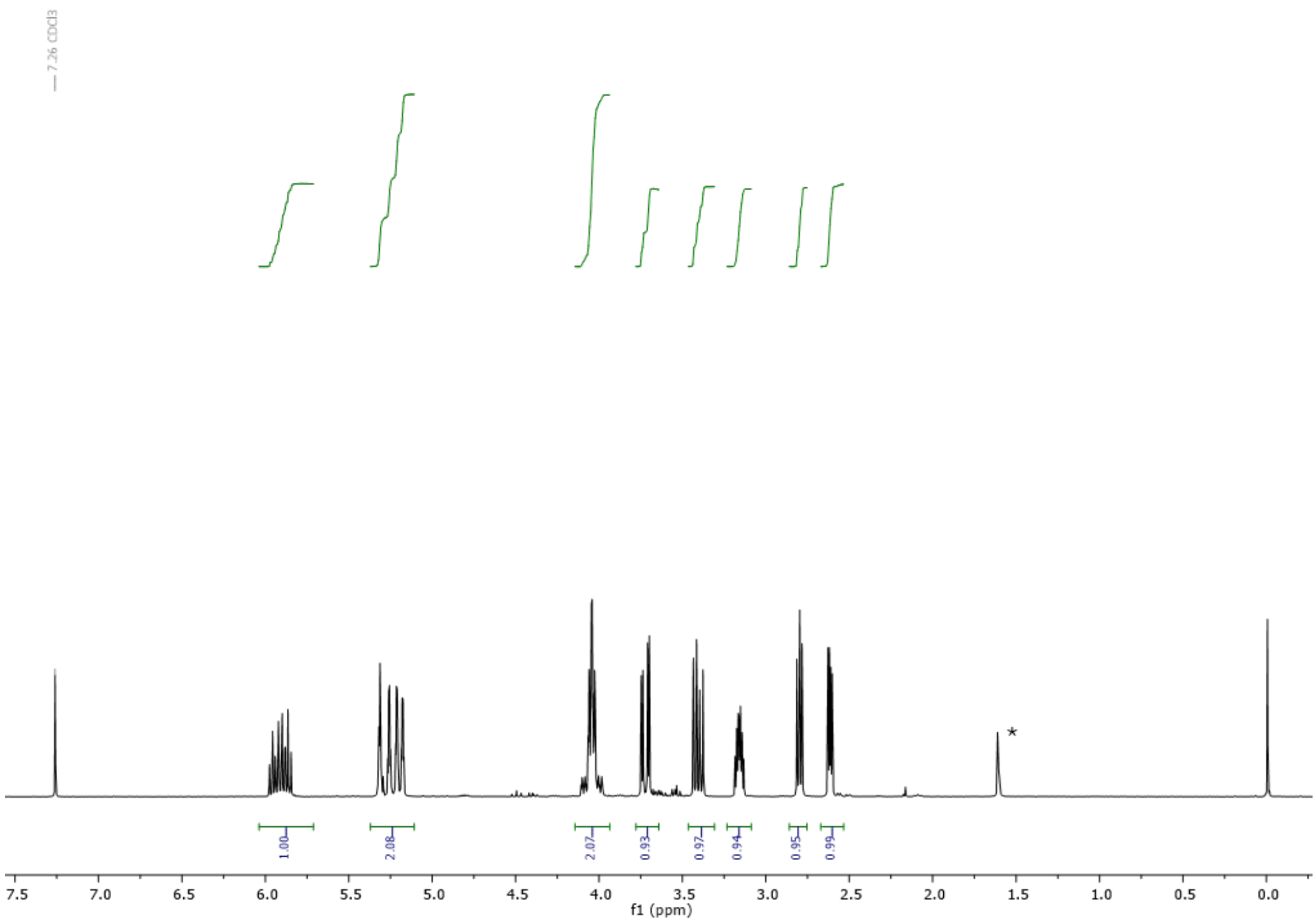

Figure ESI 14 - ^{1}H NMR of glycidyl allyl ether (GAE)

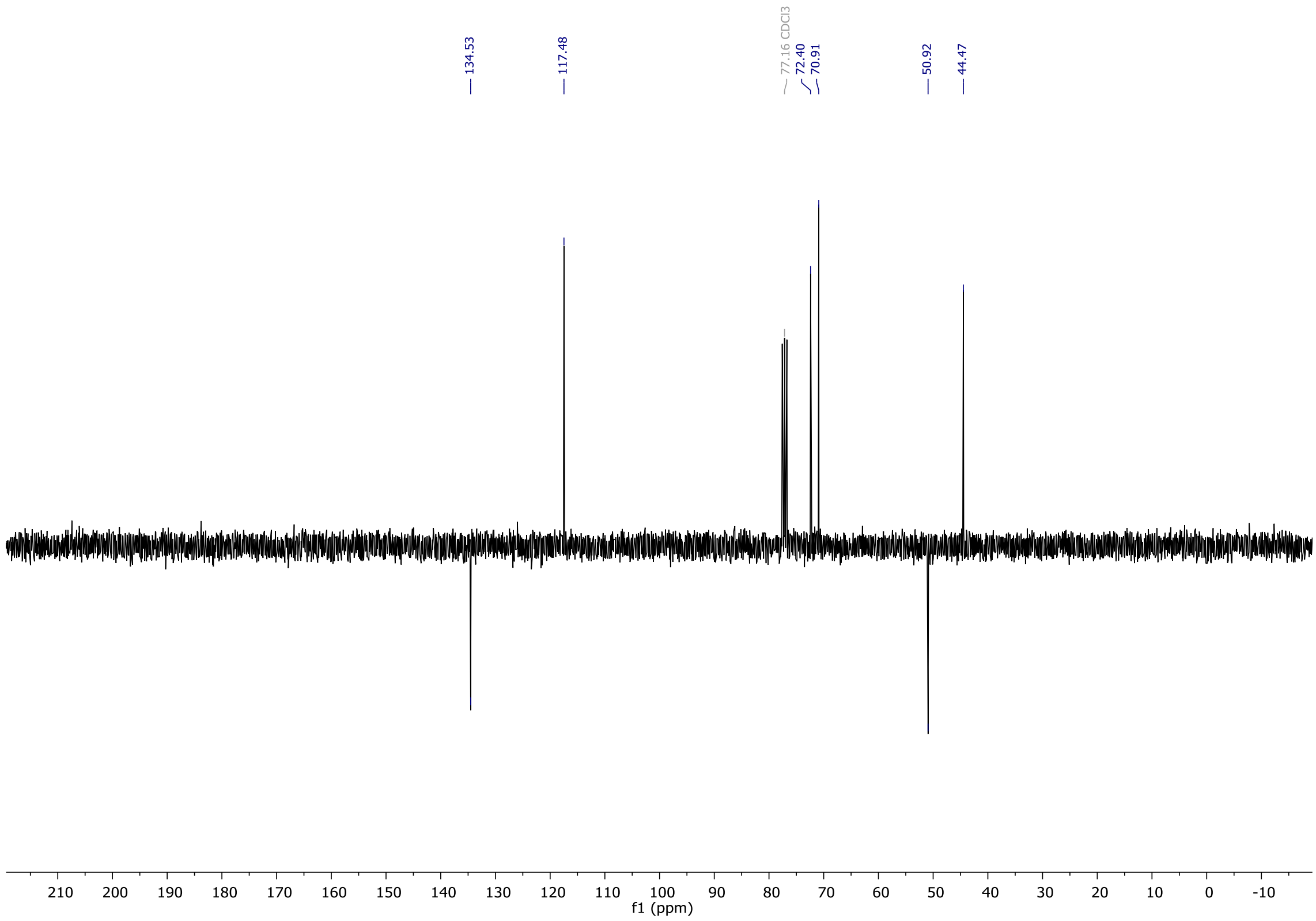

Figure ESI 15 - ^{13}C NMR of glycidyl allyl ether (GAE)

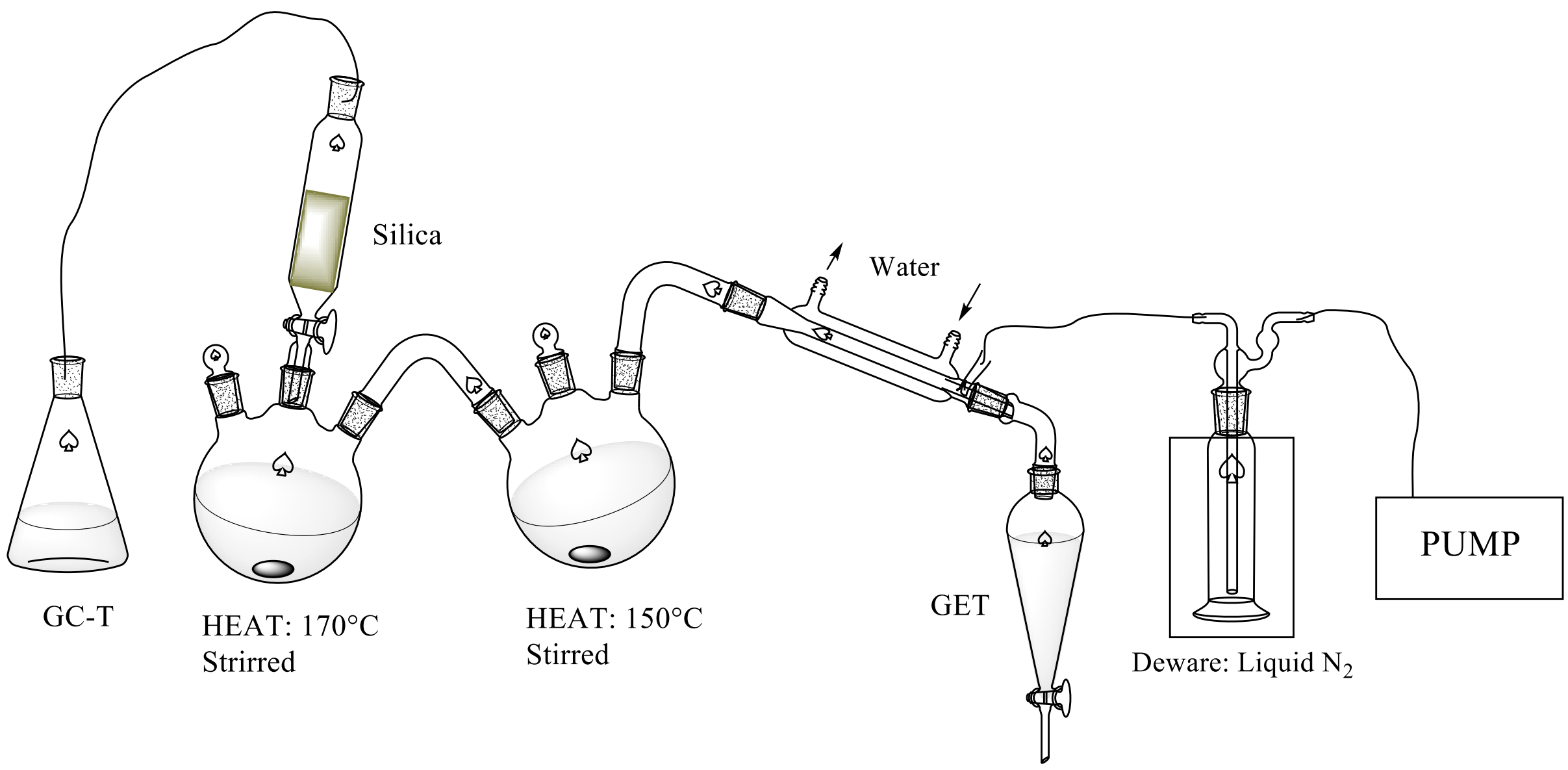

Figure ESI 16 – Decarboxylation reaction setup

5. Study on Guaiacol

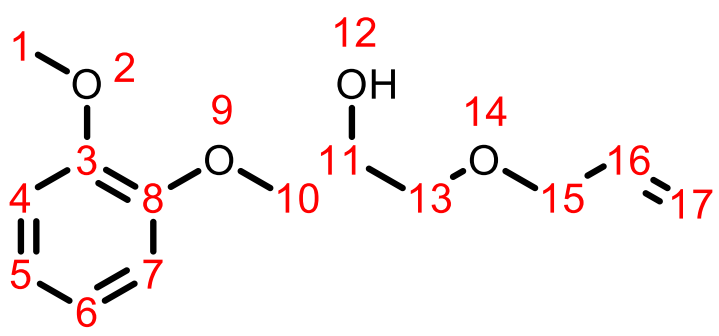

^{1}H NMR (300 MHz, Chloroform-*d*) δ 7.01 – 6.80 (m, 4H) [4, 5, 6, 7], 5.99 – 5.78 (m, 1H) [16], 5.34 – 5.08 (m, 2H) [17], 4.24 – 3.95 (m, 5H) [10, 11, 13], 3.82 (s, 3H) [1], 3.70 – 3.44 (m, 2H) [15].

^{13}C NMR (75 MHz, $CDCl_3$) δ 149.74 [3], 148.20 [8], 134.51 [16], 121.88 [6], 120.99 [7], 117.15 [17], 114.81 [5], 111.96 [4], 72.33 [15], 71.26 [10], 70.98 [13], 69.00 [11], 55.79 [1].

HRMS m/z: Calculated for $[M+H]^+$ $C_{13}H_{18}O_4$ 239.1278; Found 239.1281 (Δm = 0.0003 m/z)

Isolated product obtained by column chromatography on silica gel with petroleum ether/ethyl acetate 80 : 20 eluant – 65% isolated yield

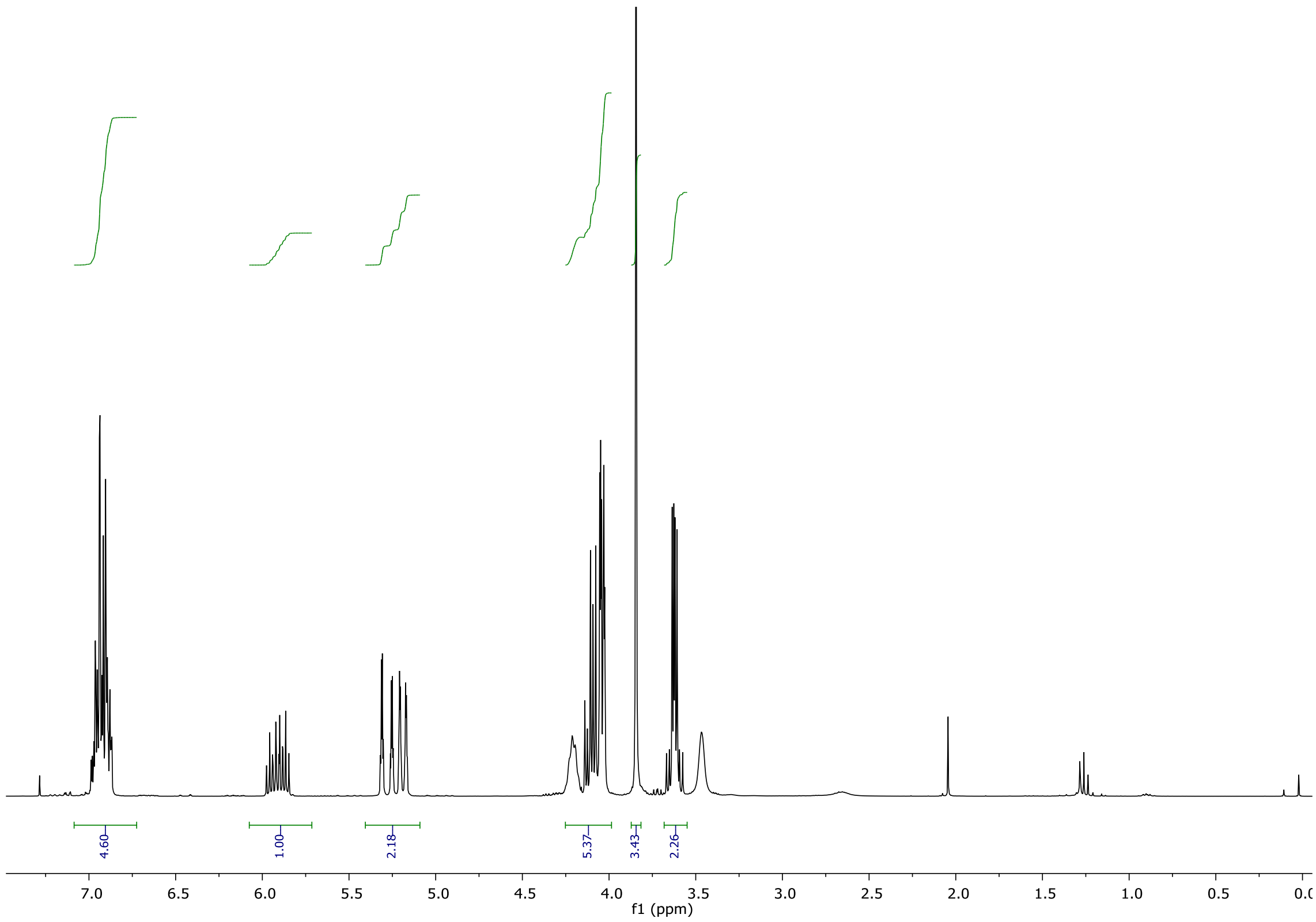

Figure ESI 17 - ^{1}H NMR of glycidyl allyl ether grafted on guaiacol

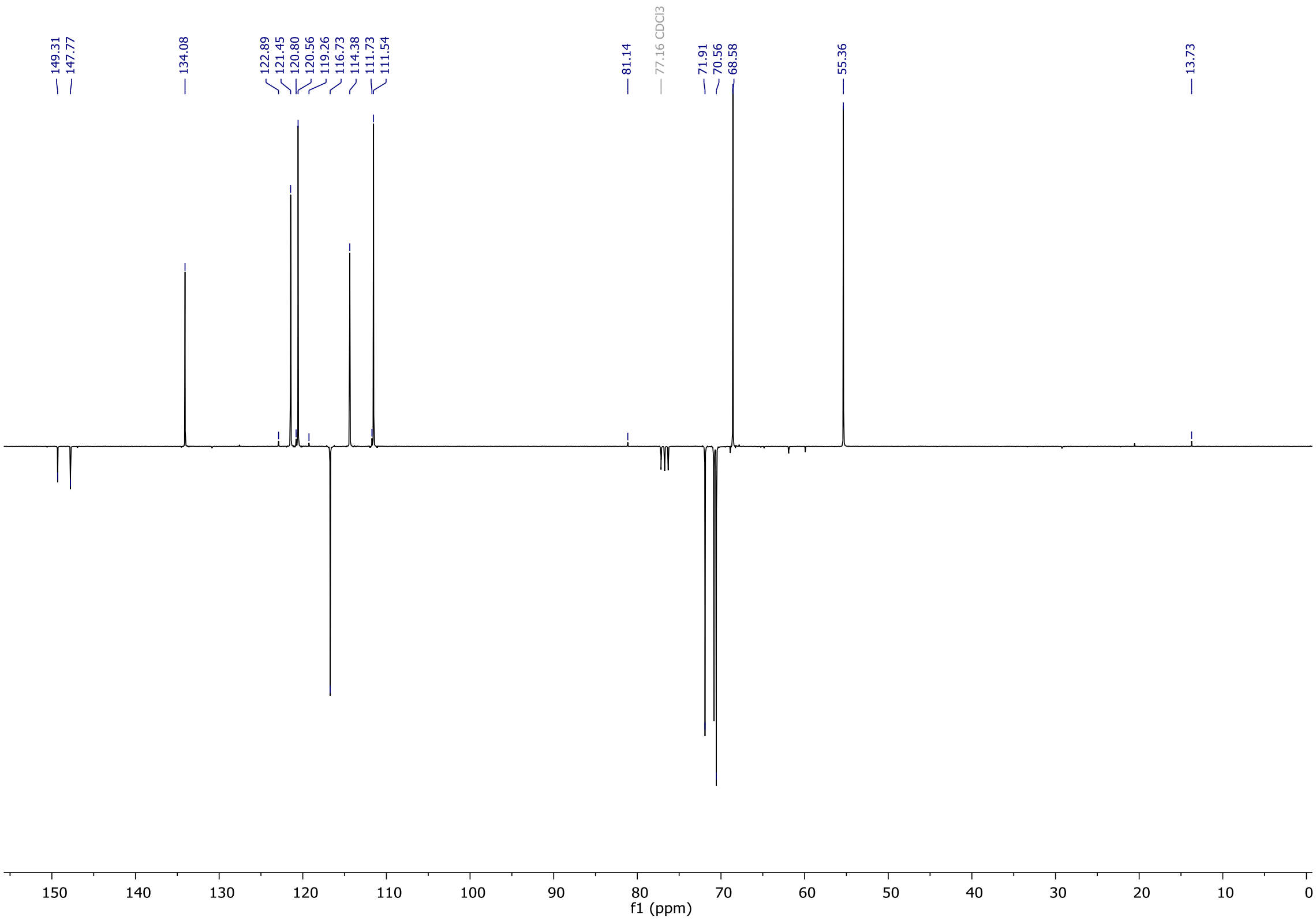

Figure ESI 18 - ^{13}C NMR of glycidyl allyl ether grafted on guaiacol

6. Functionalisation of lignin with glycidyl allyl ether

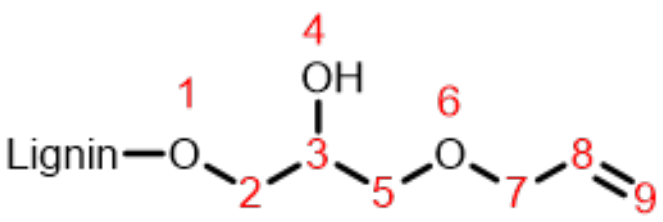

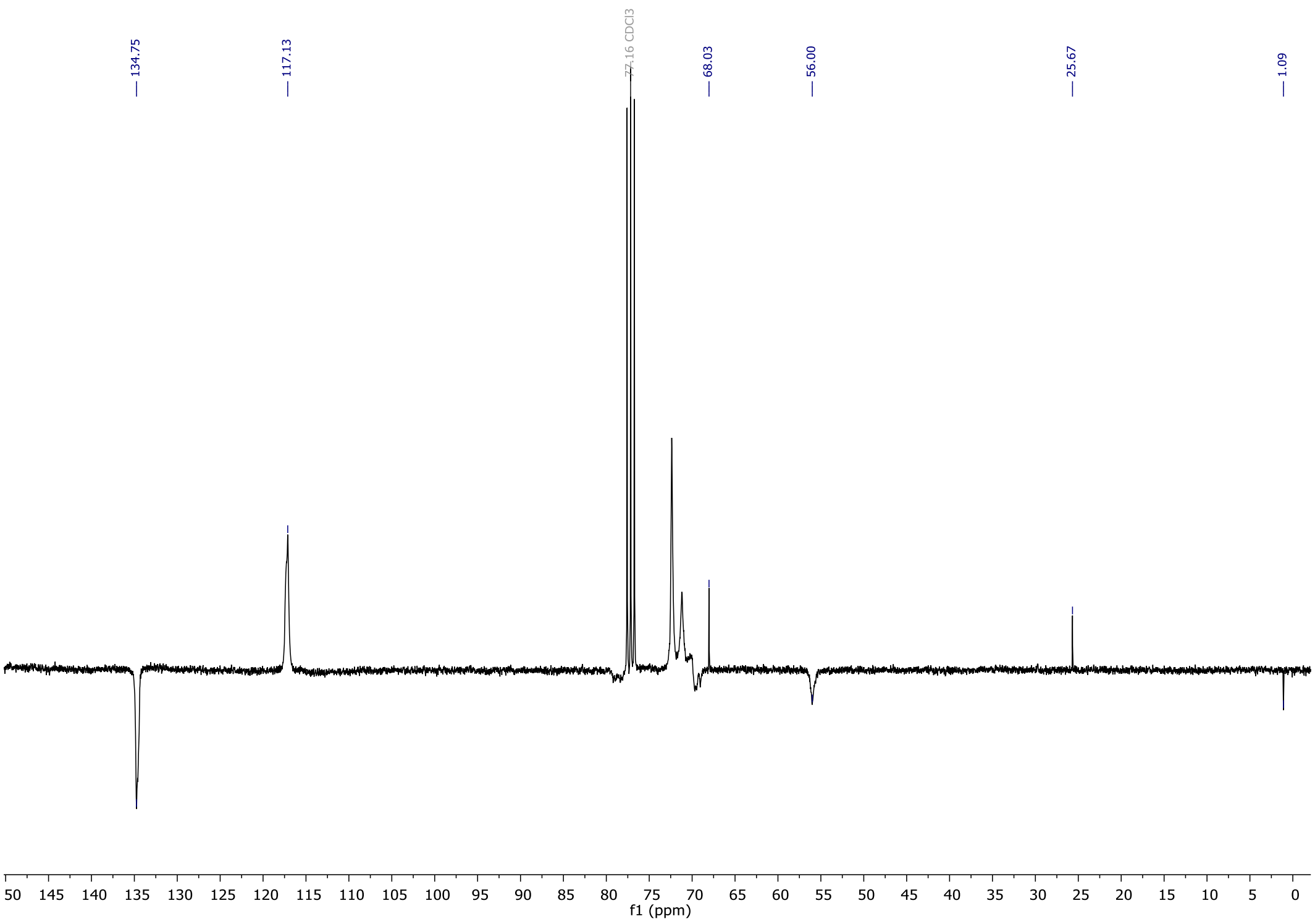

Figure ESI 19 - ^{13}C NMR of glycidyl allyl ether grafted on kraft lignin

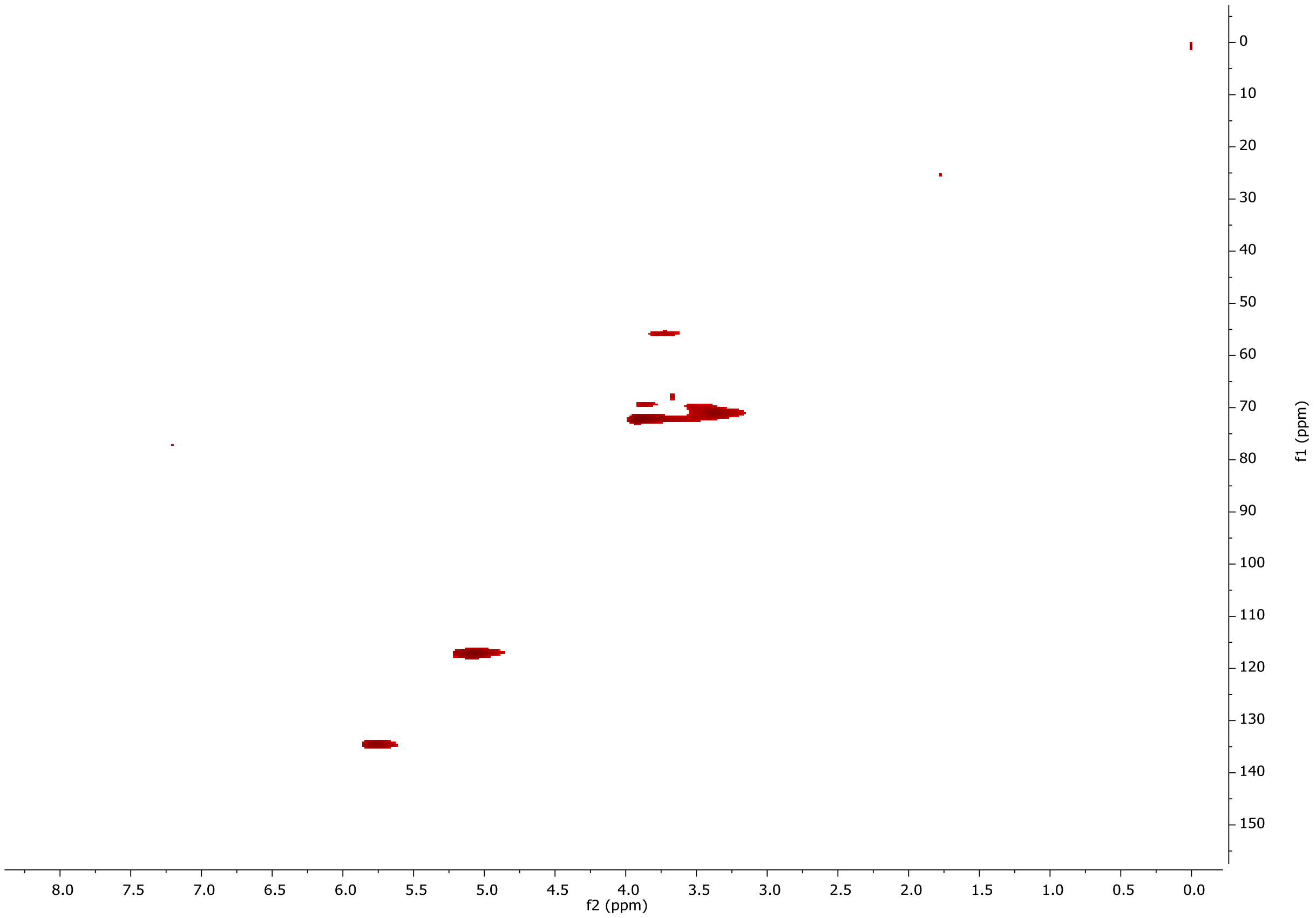

Figure ESI 20 - HSQC of glycidyl allyl ether grafted on kraft lignin

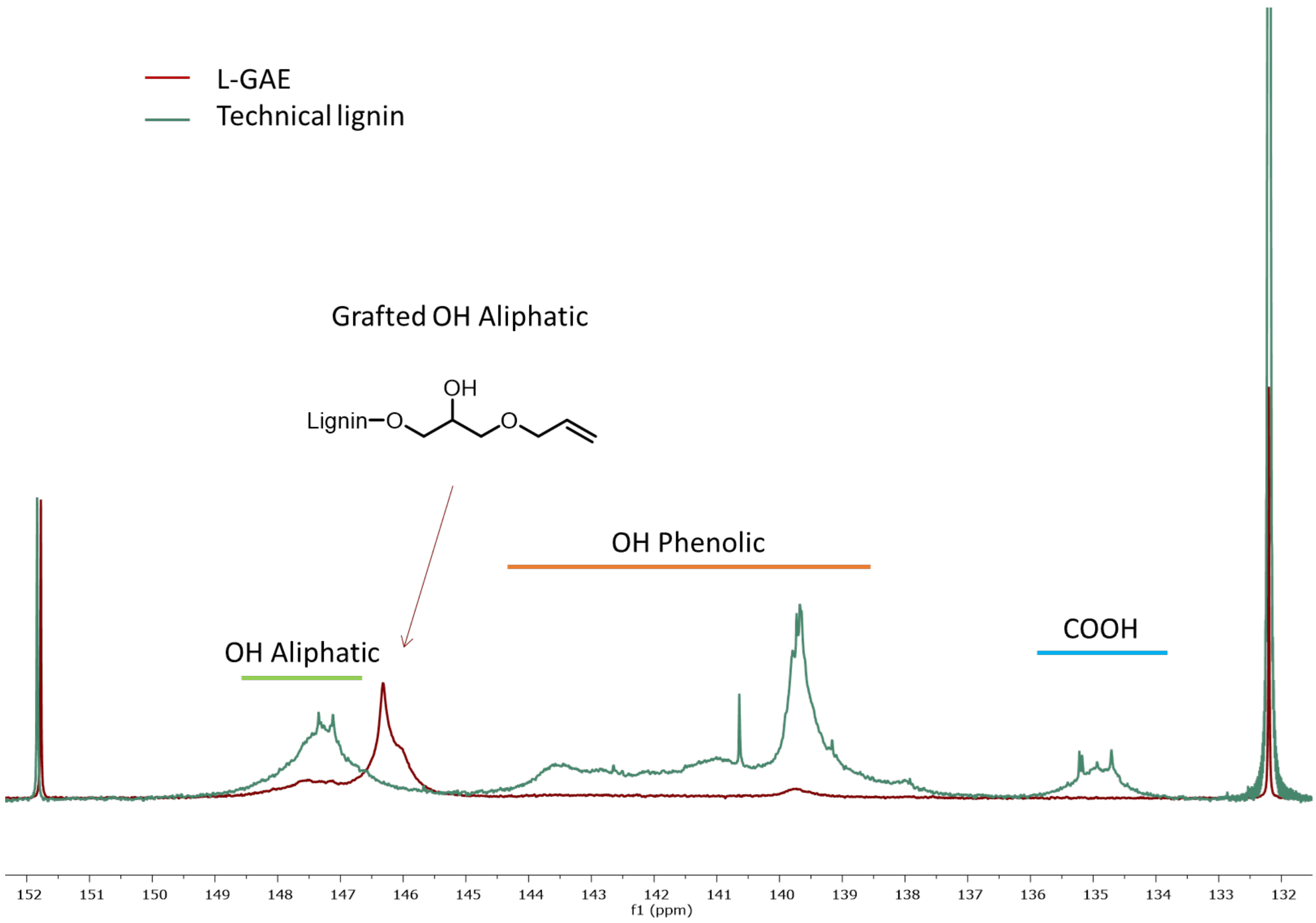

Figure ESI 21 - ^{31}P NMR of glycidyl allyl ether grafted on kraft lignin

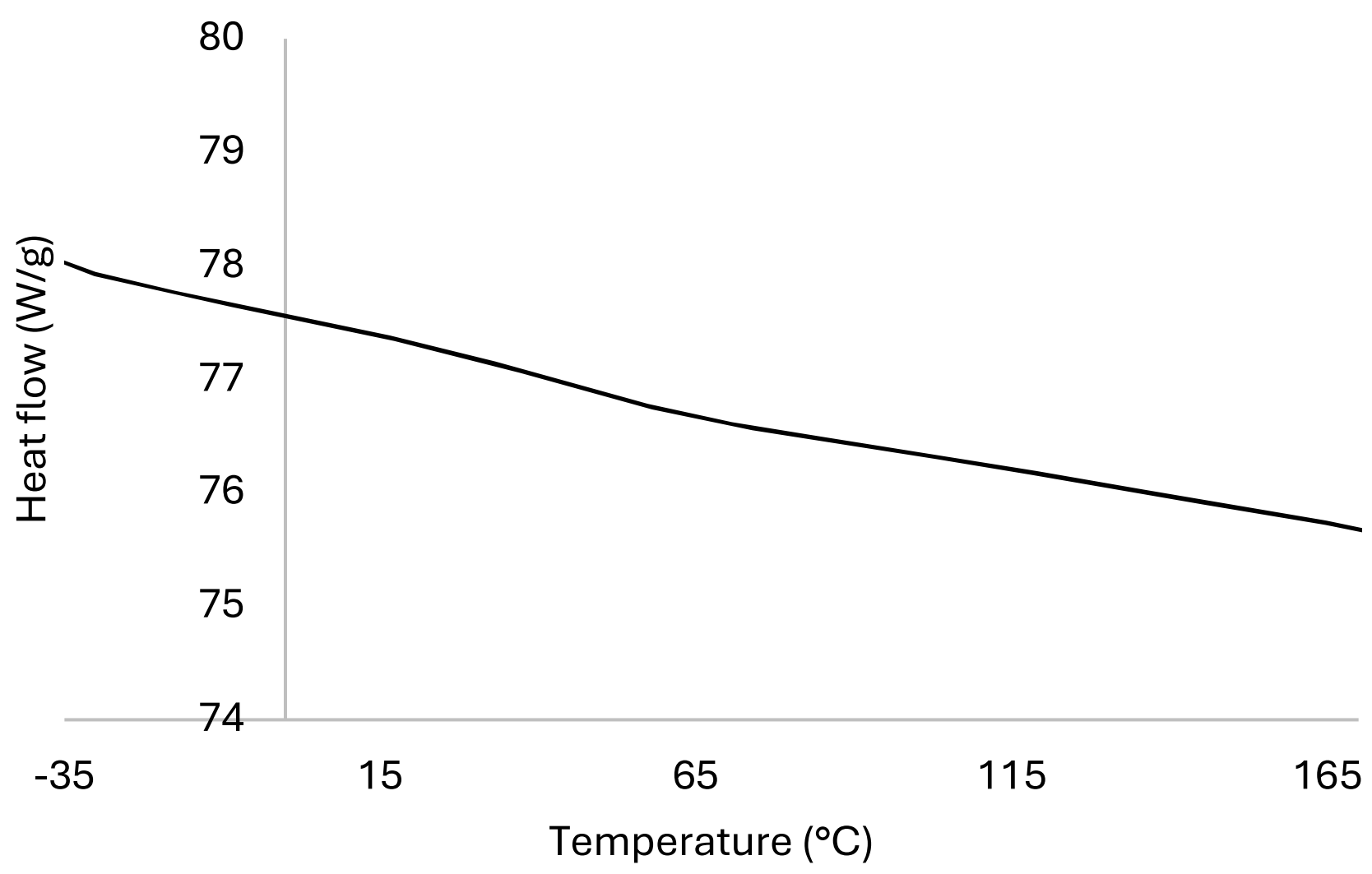

Figure ESI 22 - DSC of glycidyl allyl ether grafted on kraft lignin (endo down)

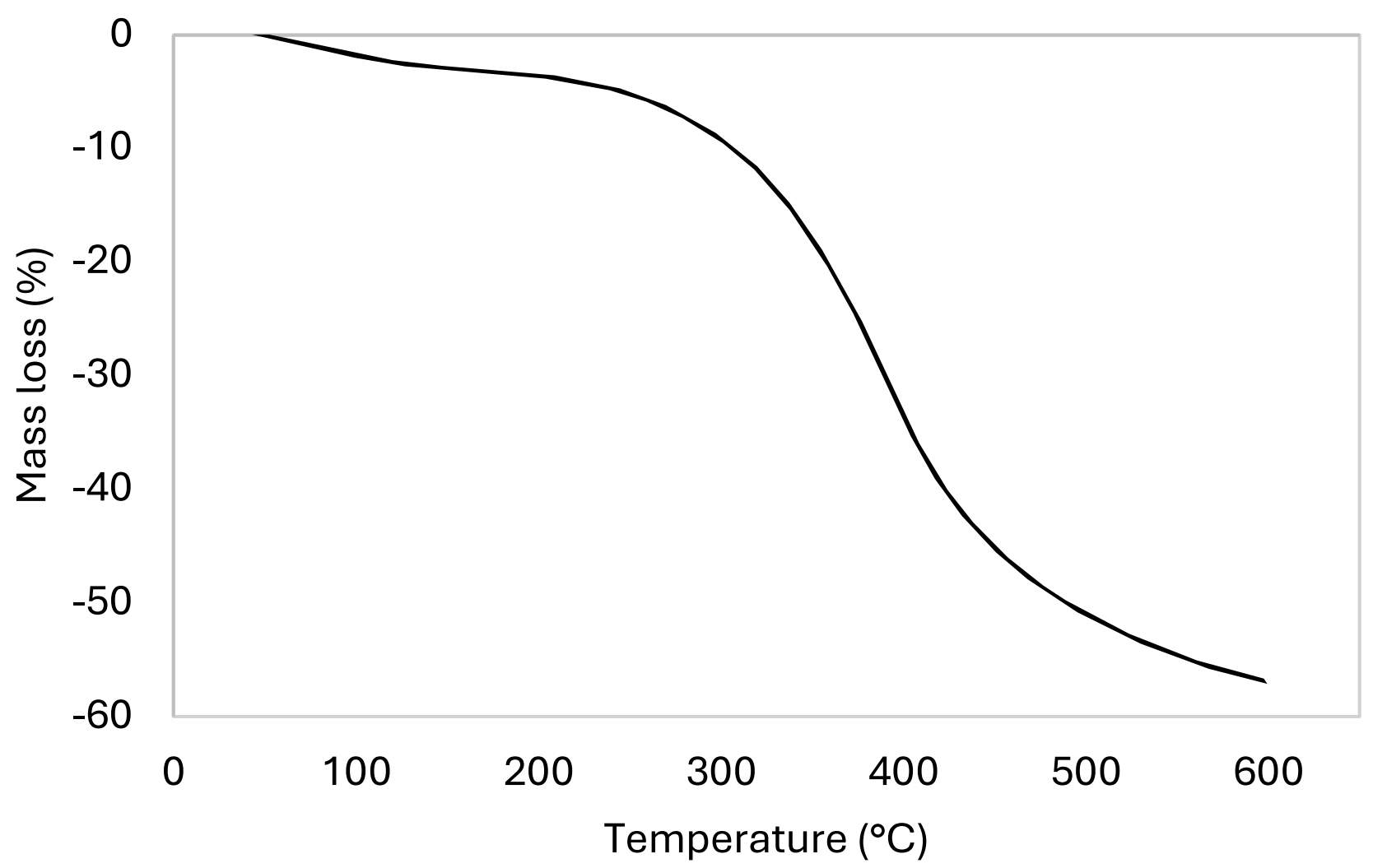

Figure ESI 23 - TGA of glycidyl allyl ether grafted on kraft lignin

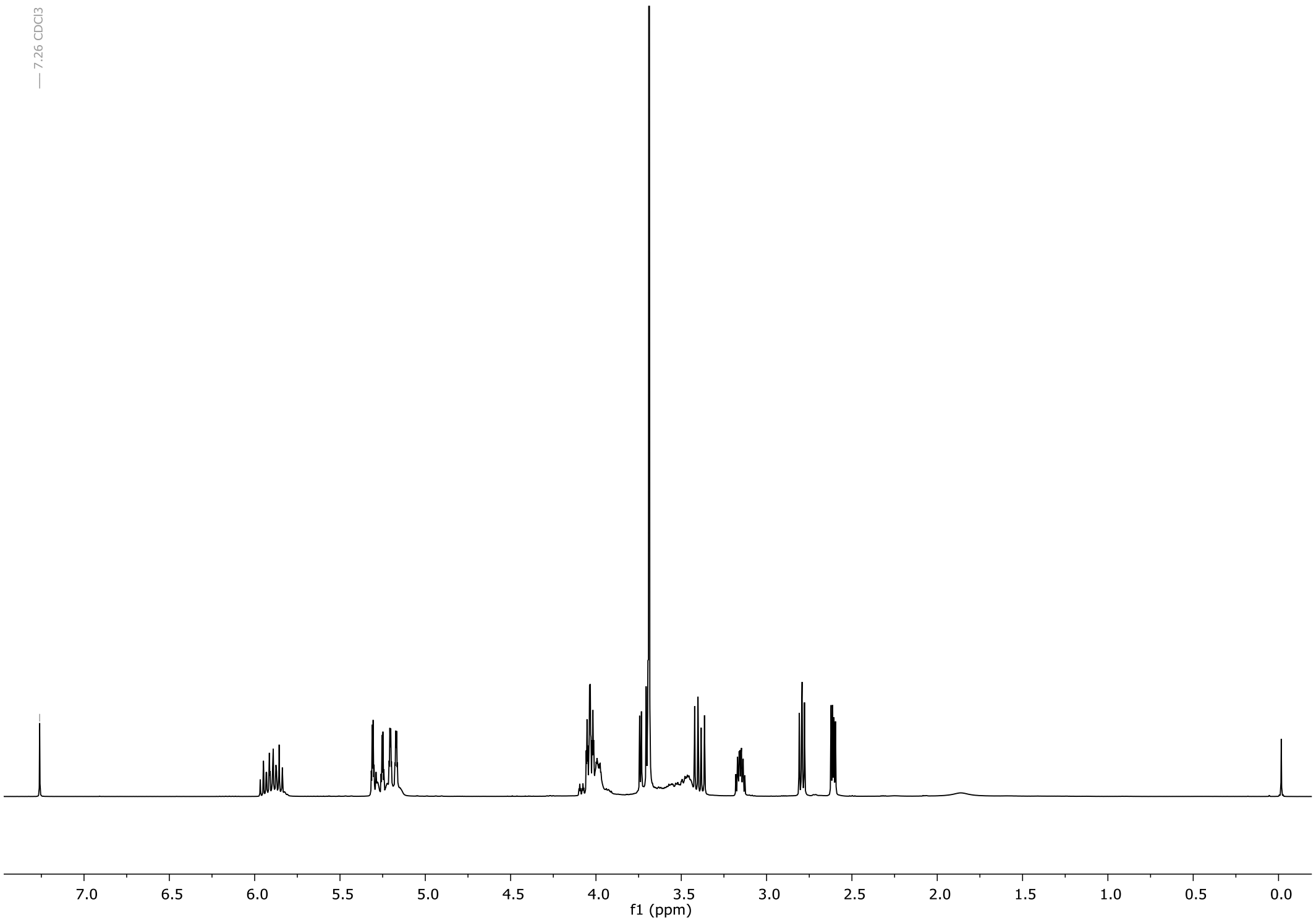

Figure ESI 24 - ¹H NMR of GAE after reaction without lignin

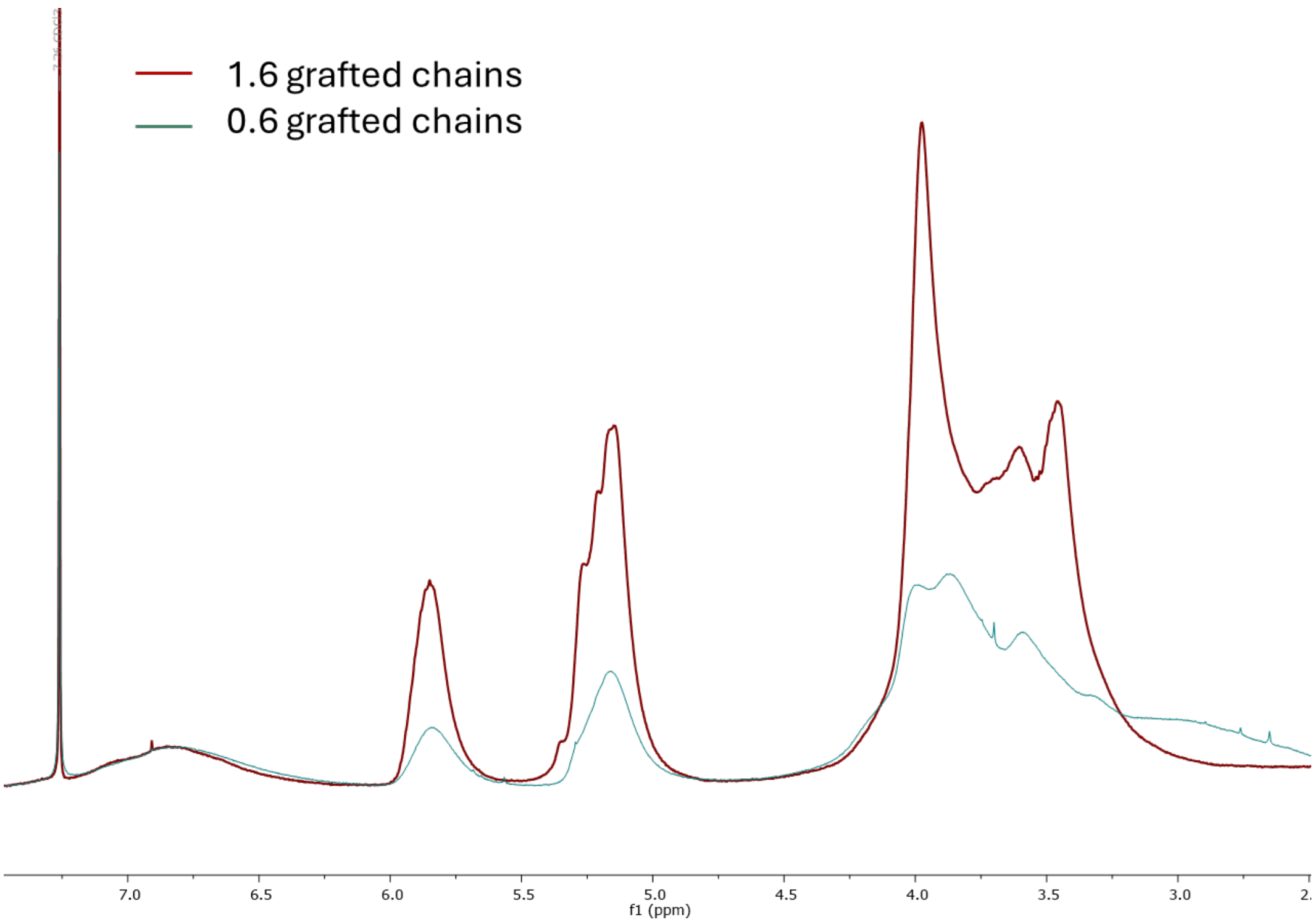

Figure ESI 25 - Comparison of ¹H NMR of L-GAE with 1.6 grated chains (red) and L-GAE with 0.6 grafted chains (green)

7. Functionalisation with different types of lignin with GAE

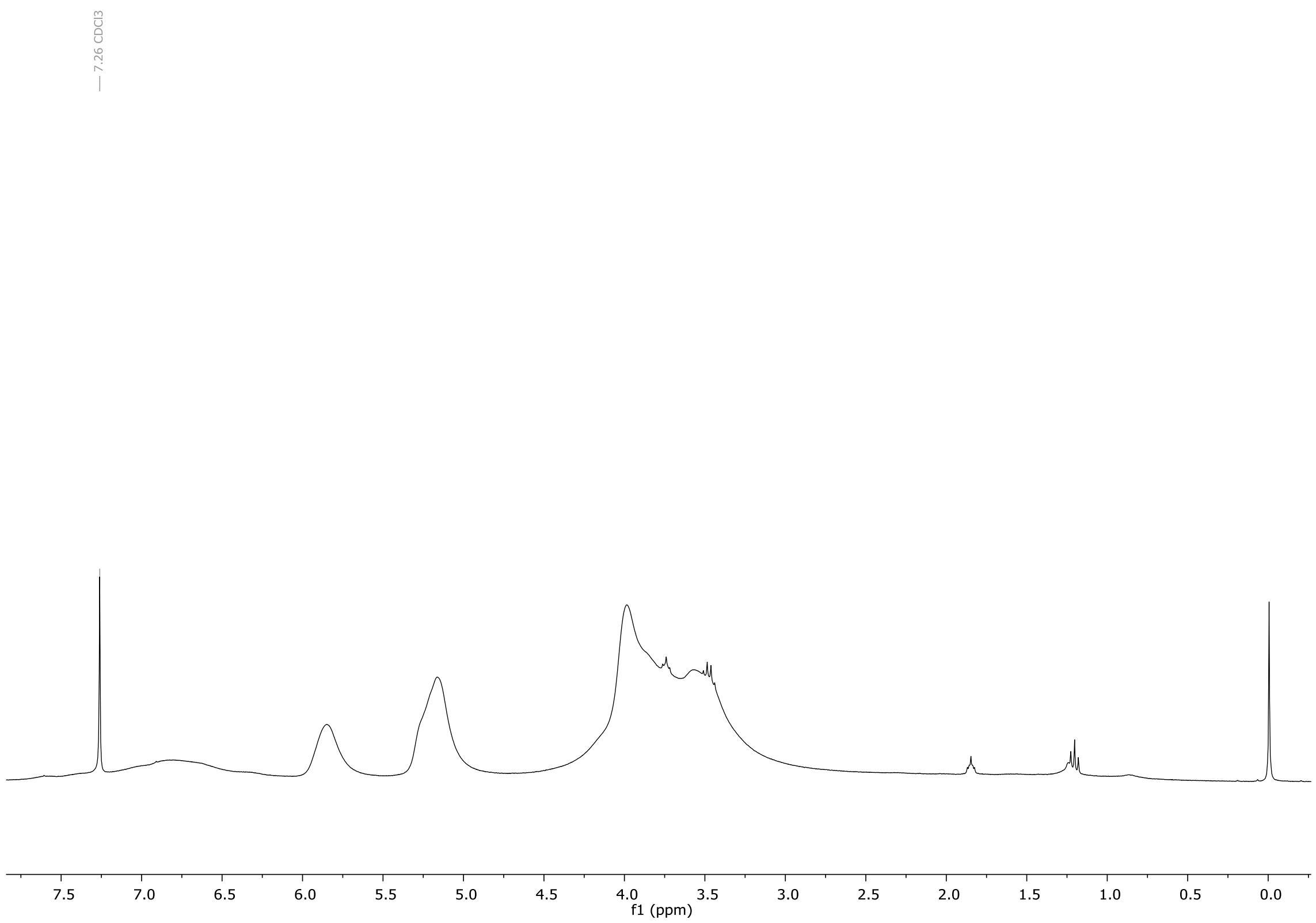

Figure ESI 26 - ^{1}H NMR of GAE grafted on Organosolv lignin

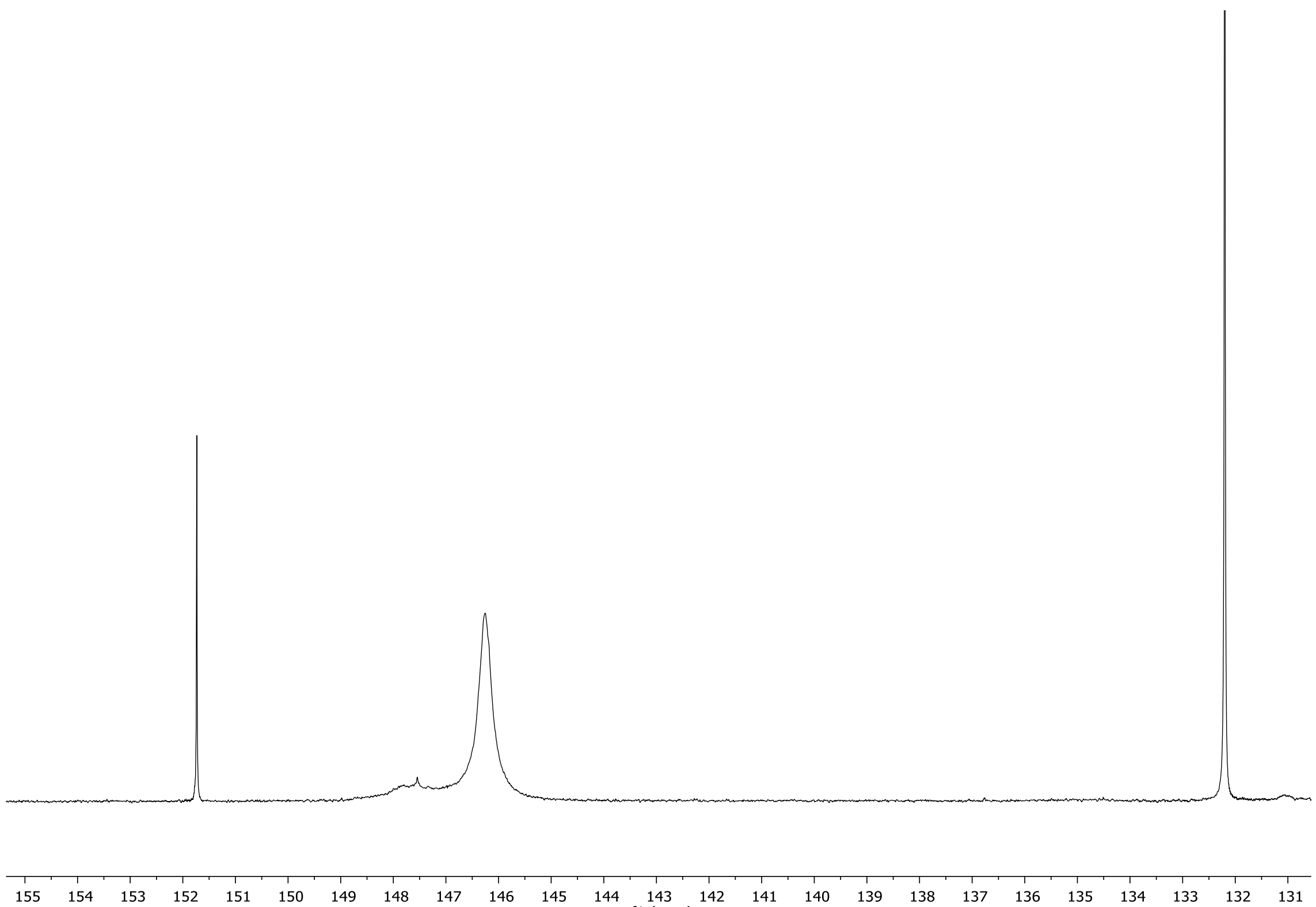

Figure ESI 27 - ^{31}P NMR of GAE grafted on Organosolv lignin

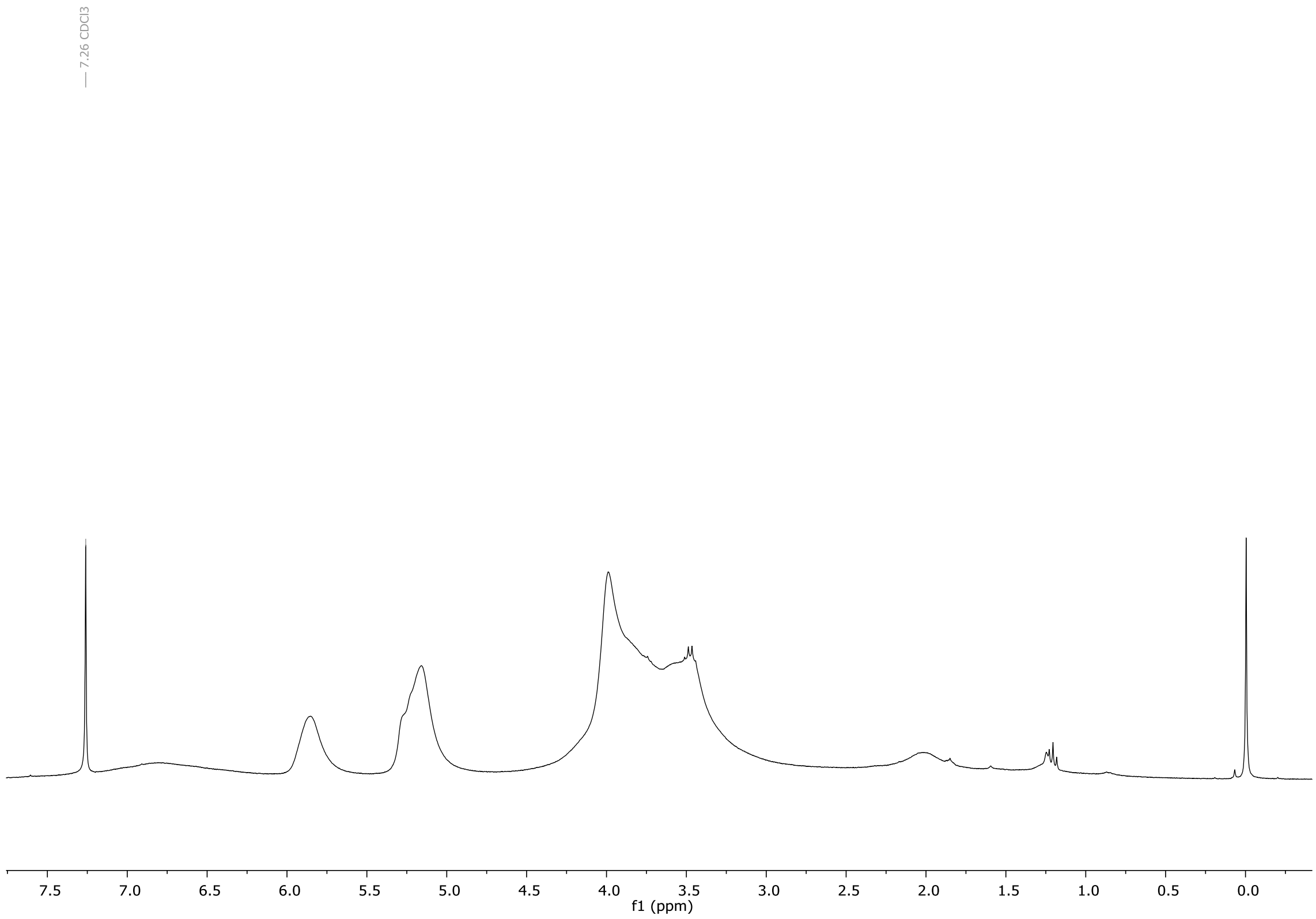

Figure ESI 28 - ^{1}H NMR spectra of GAE grafted on Soda lignin

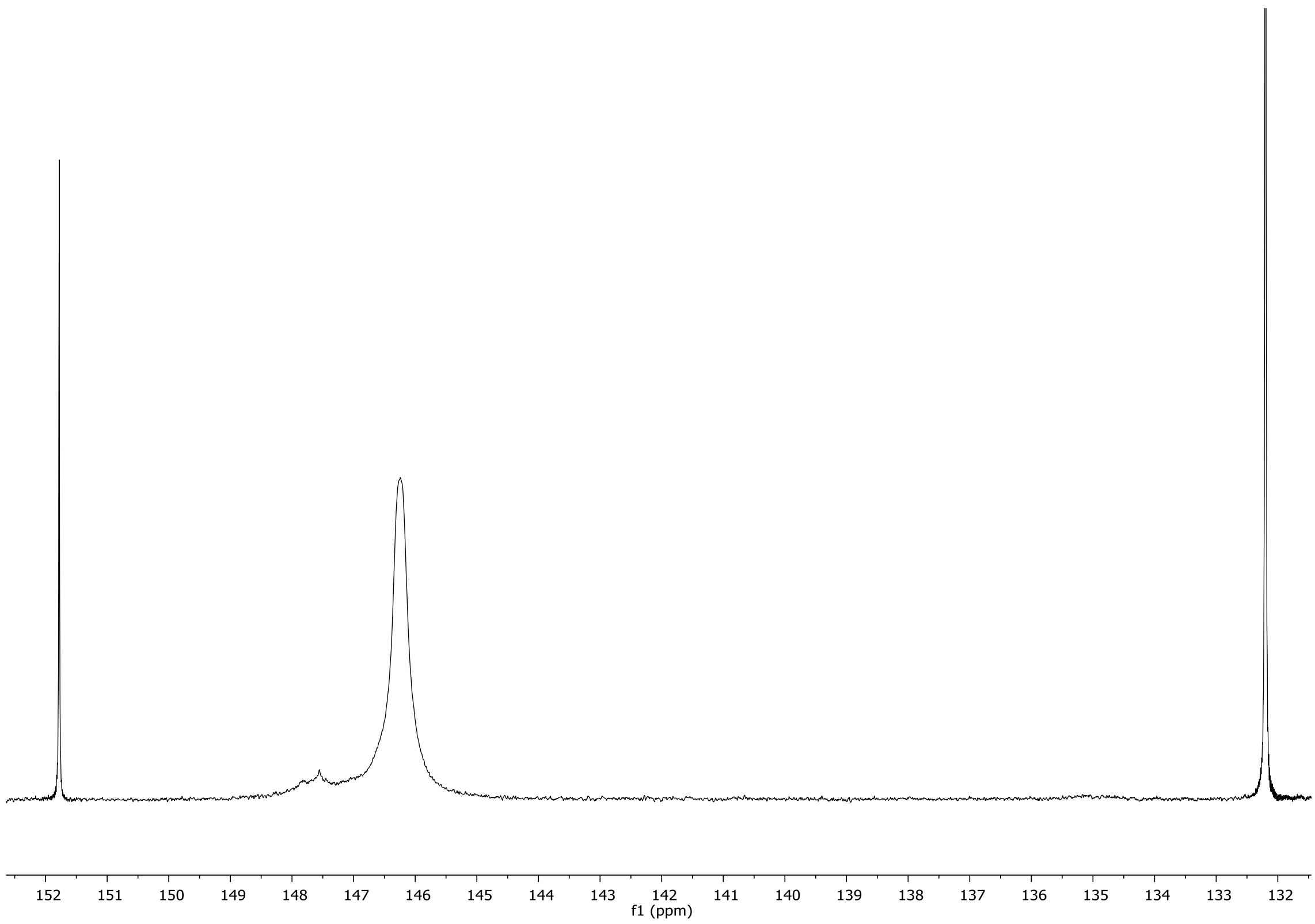

Figure ESI 29 - ^{31}P NMR spectra of GAE grafted on Soda lignin

8. Functionalisation of lignin with GTE

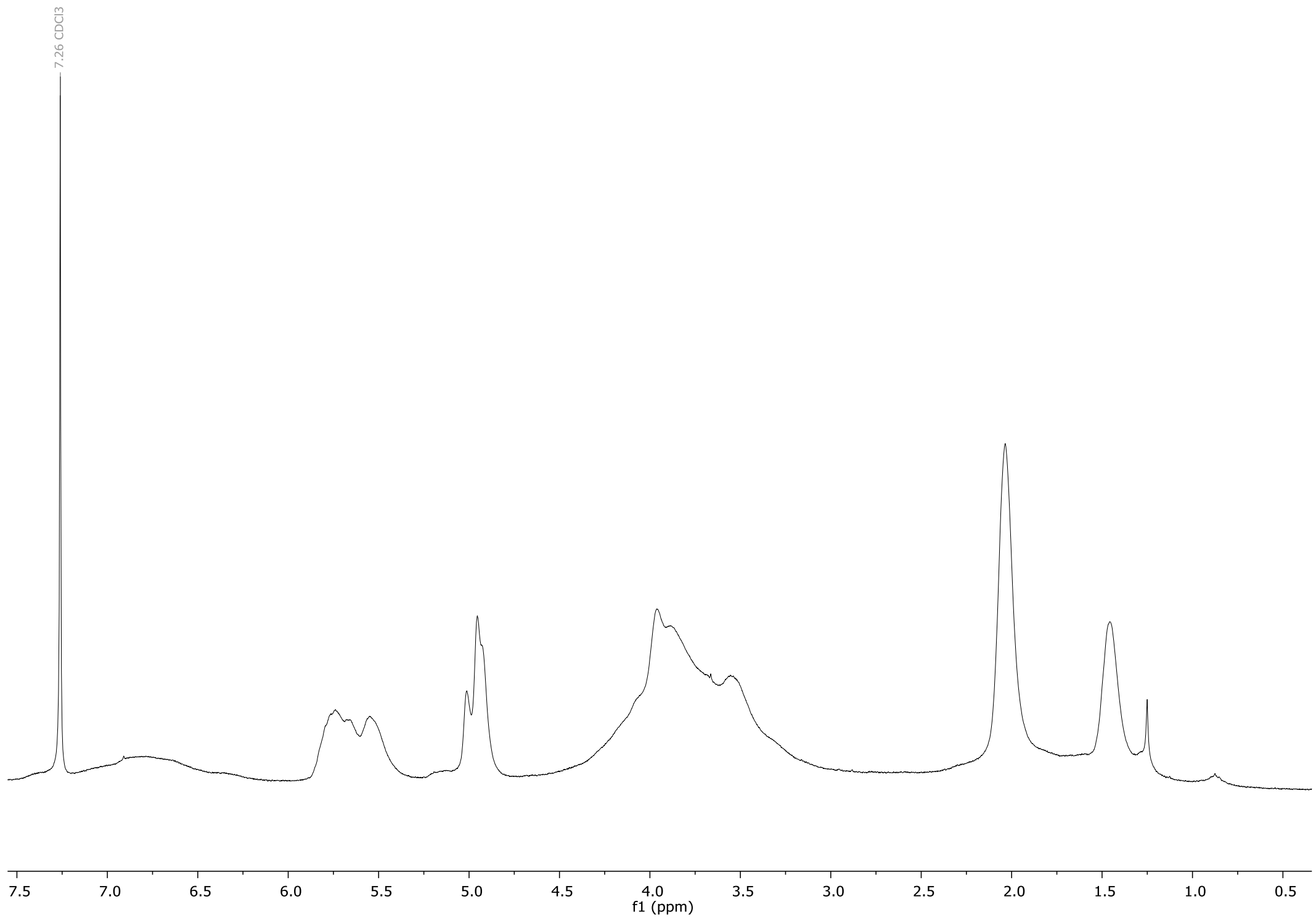

Figure ESI 30 : ^{1}H NMR of GTE grafted on Kraft lignin

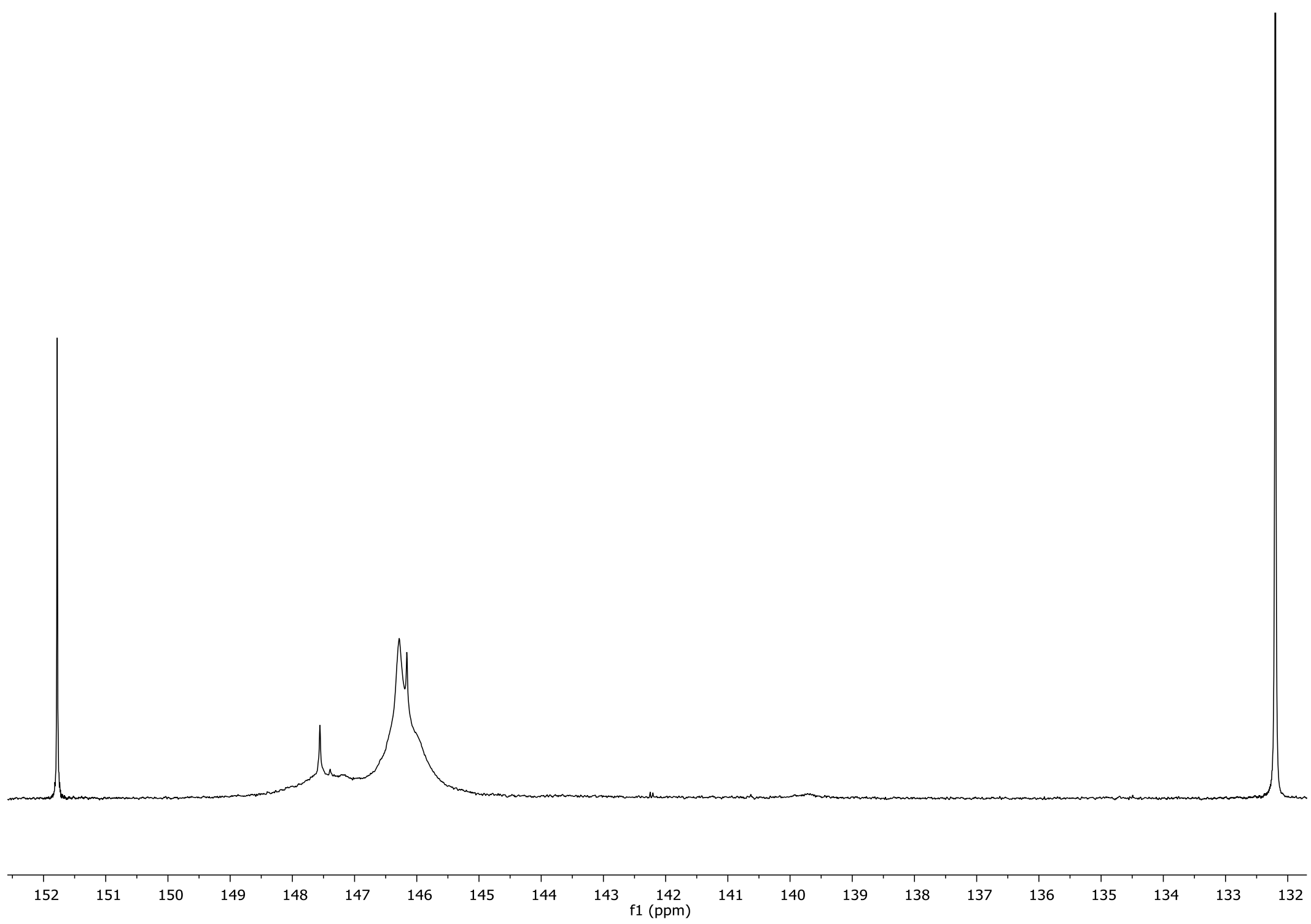

Figure ESI 31 - ^{31}P NMR of GTE grafted on Kraft lignin

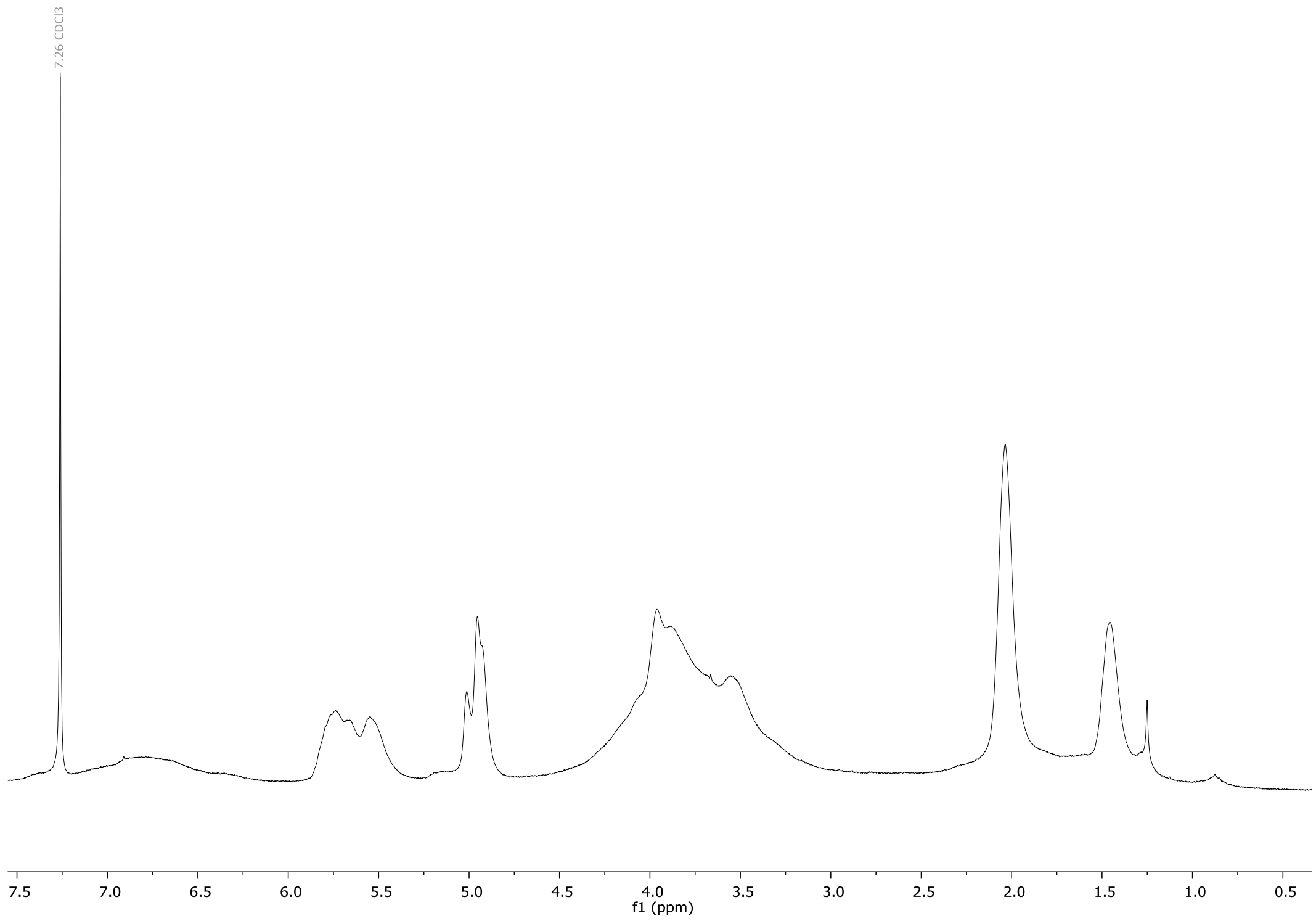

Figure ESI 32 : ^{1}H NMR of GTE grafted on Organosolv lignin

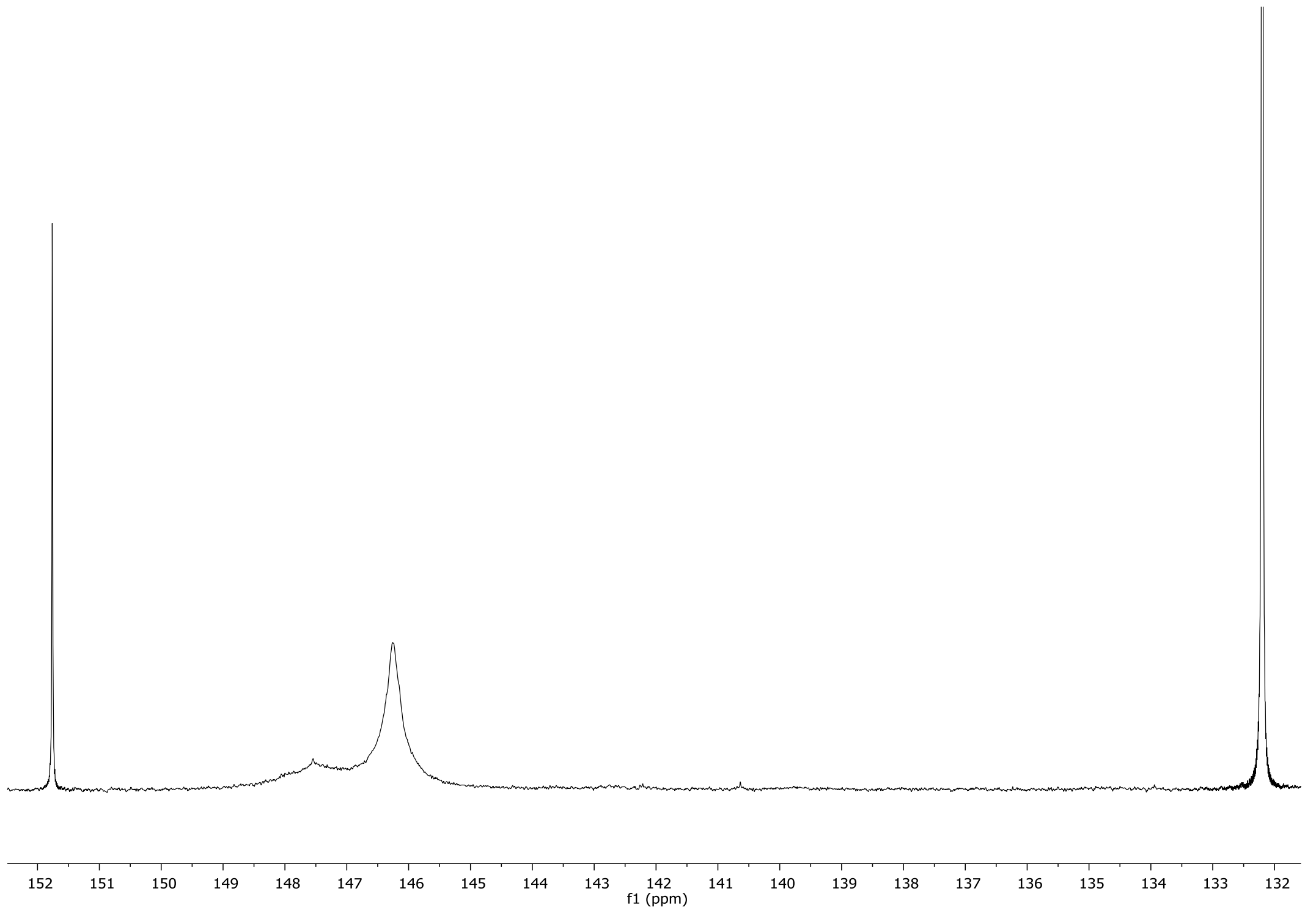

Figure ESI 33 - ^{31}P NMR of GTE grafted on Organosolv lignin

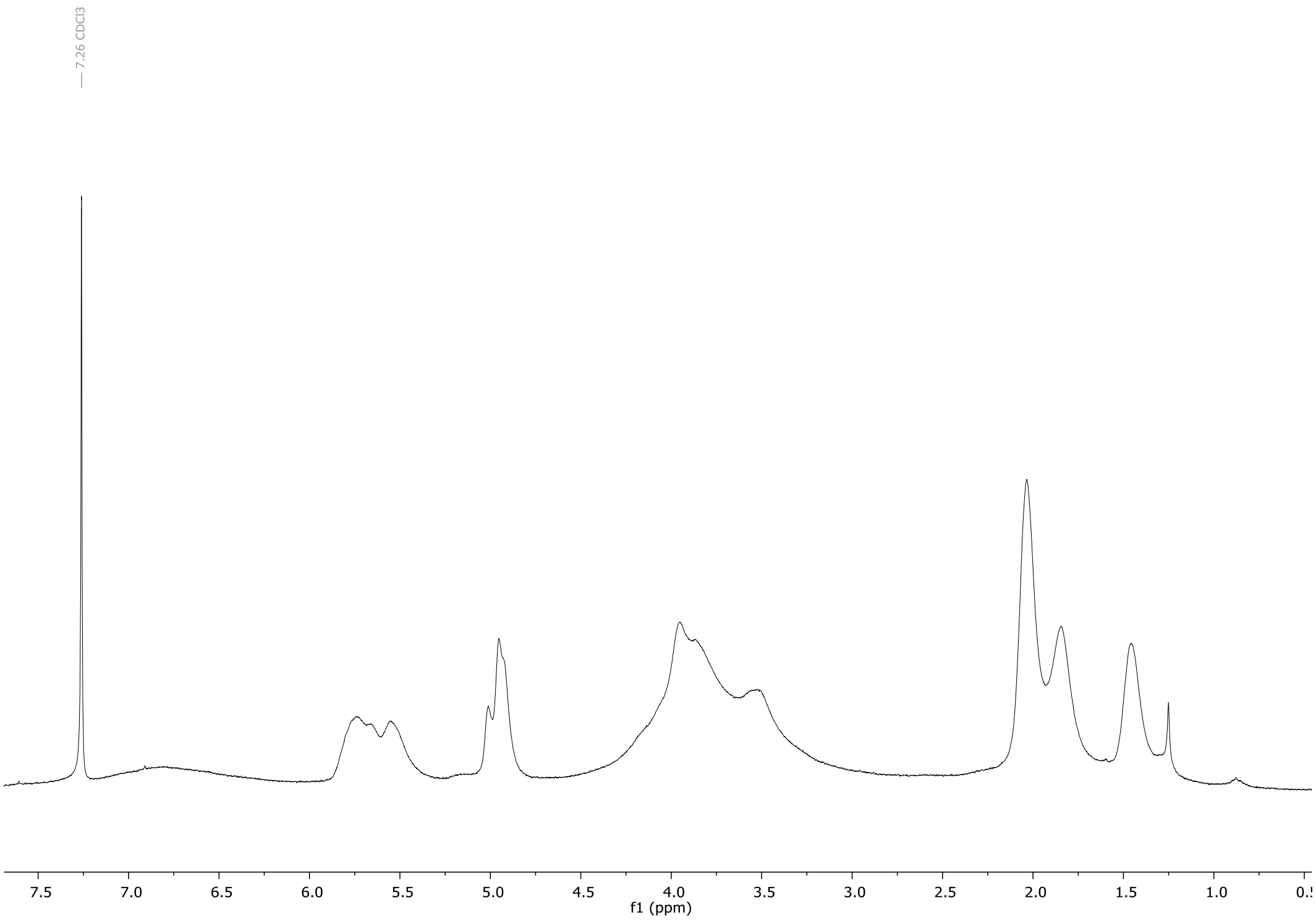

Figure ESI 34 : ^{1}H NMR of GET grafted on Soda lignin

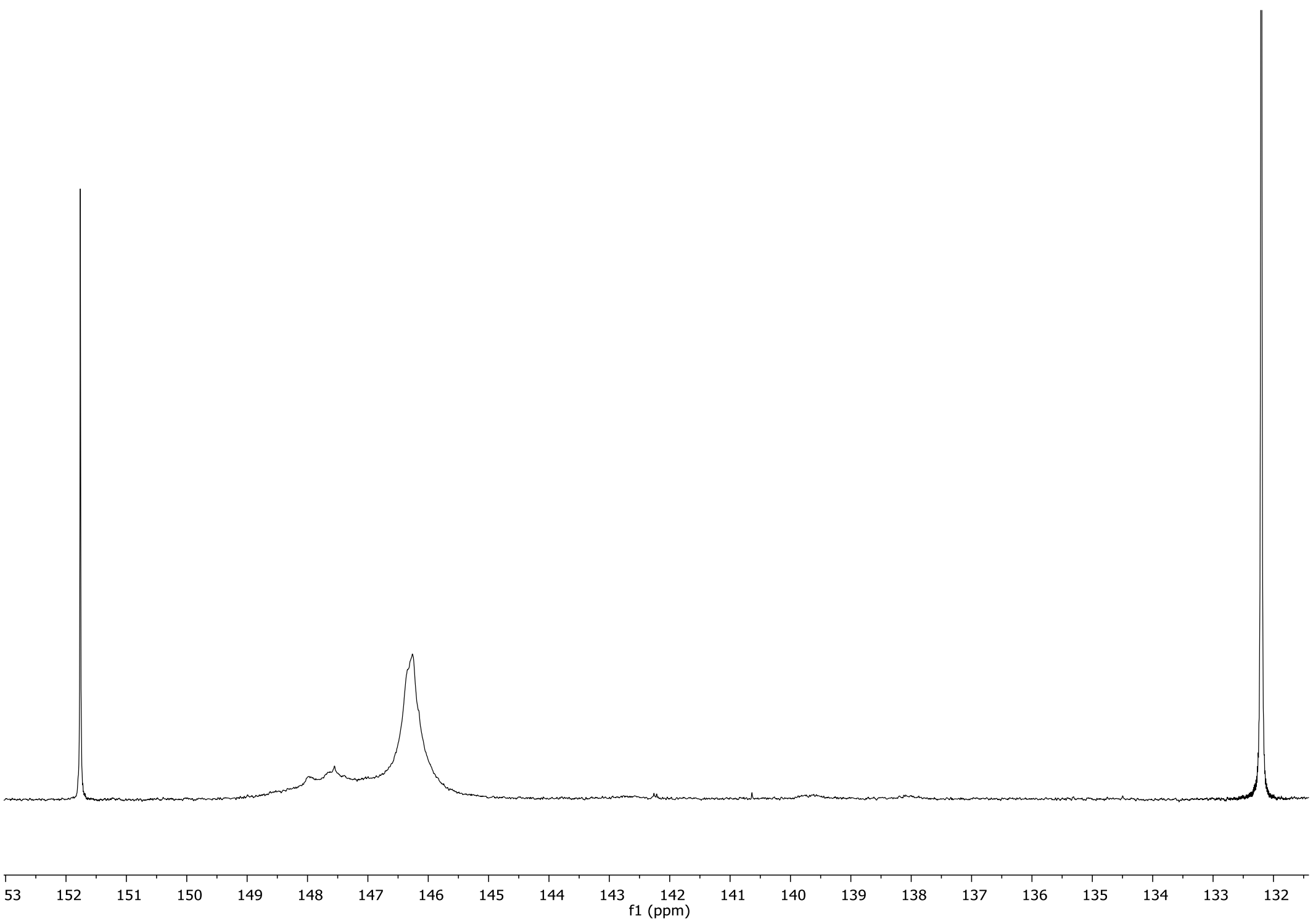

Figure ESI 35 - ^{31}P NMR of GTE grafted on Soda lignin

9. TGA comparison of Kraft and modified lignins

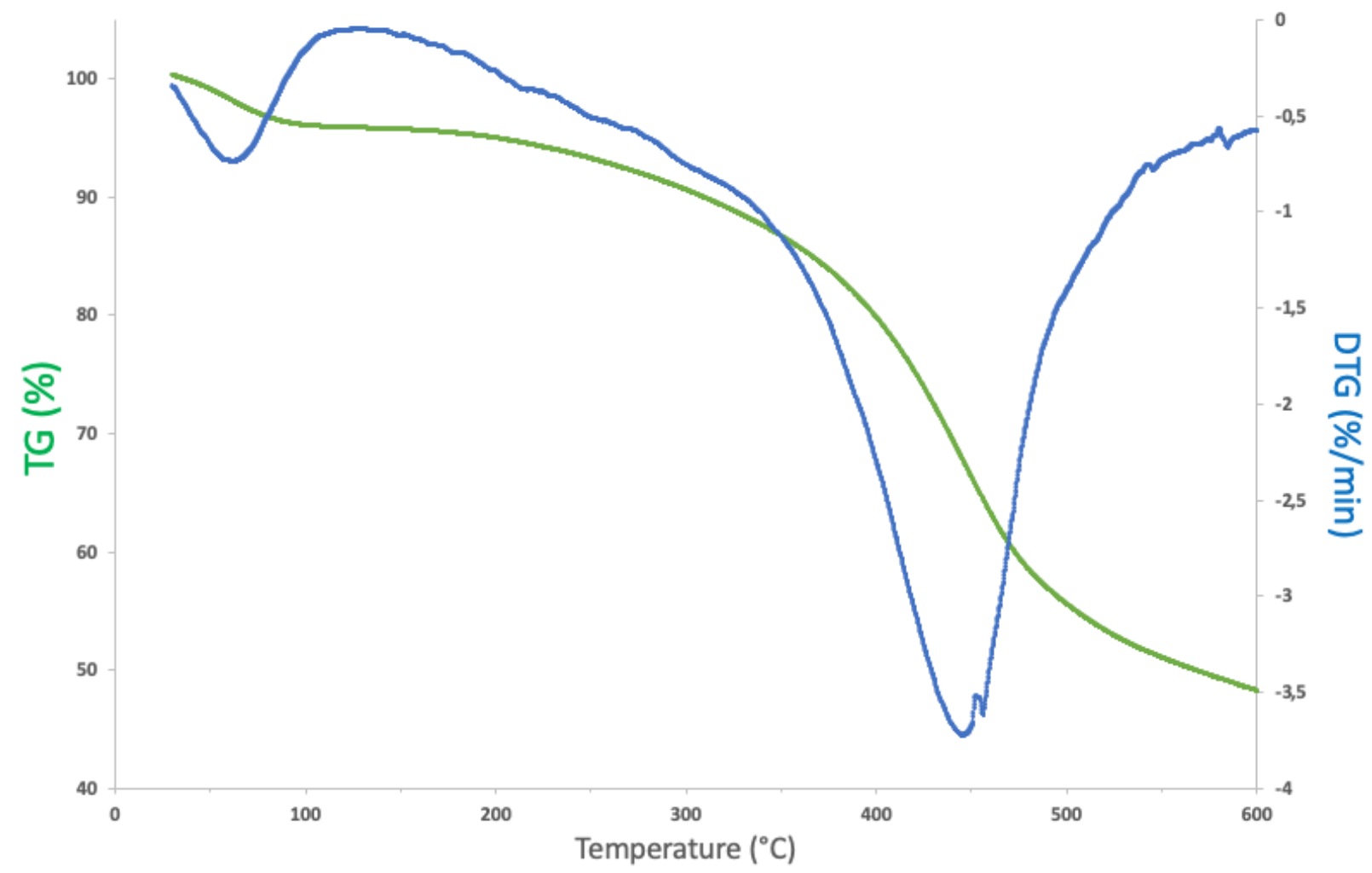

Figure ESI 36 – TGA of technical Kraft Lignin

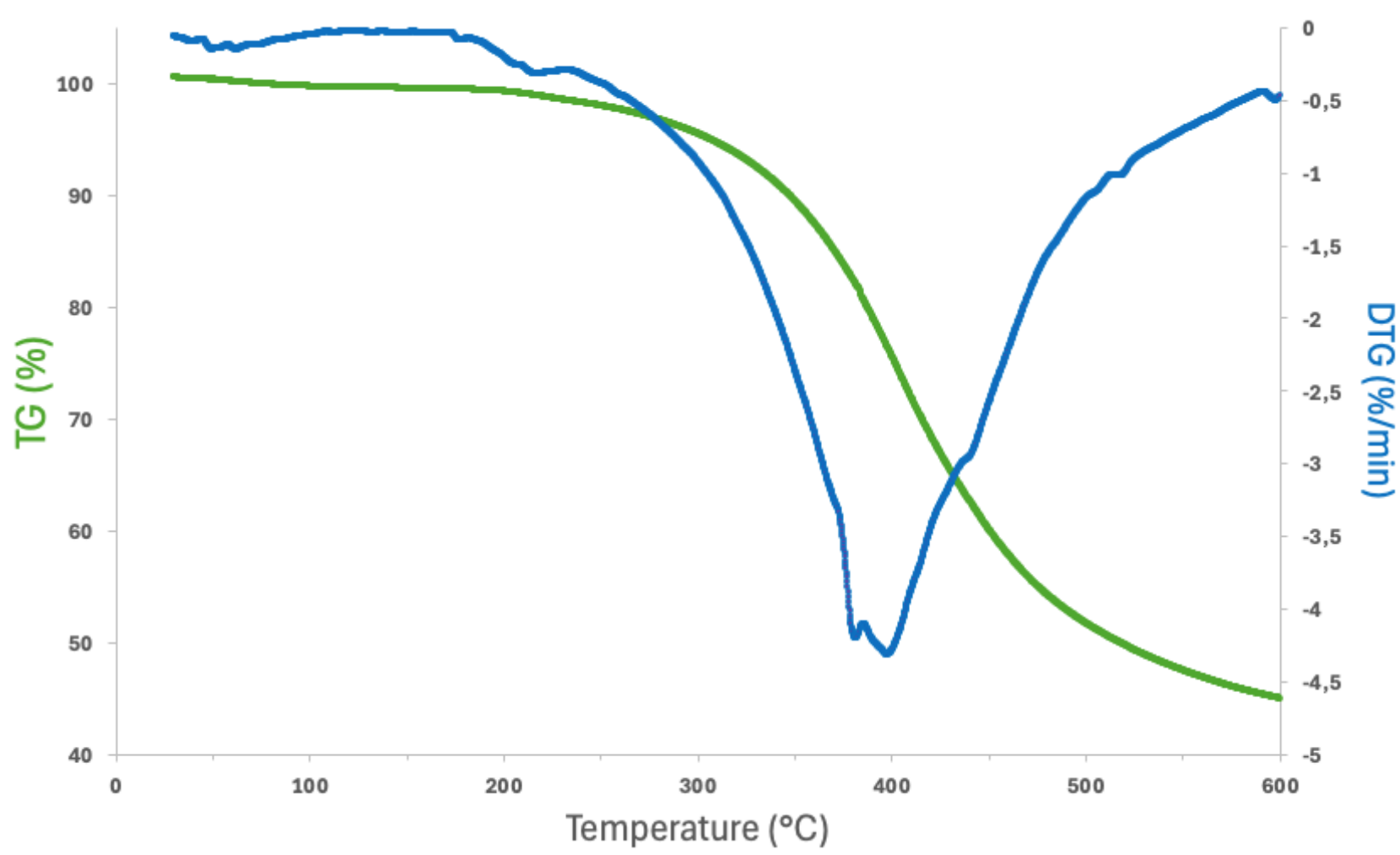

Figure ESI 37 – TGA of GAE Grafted on Kraft Lignin

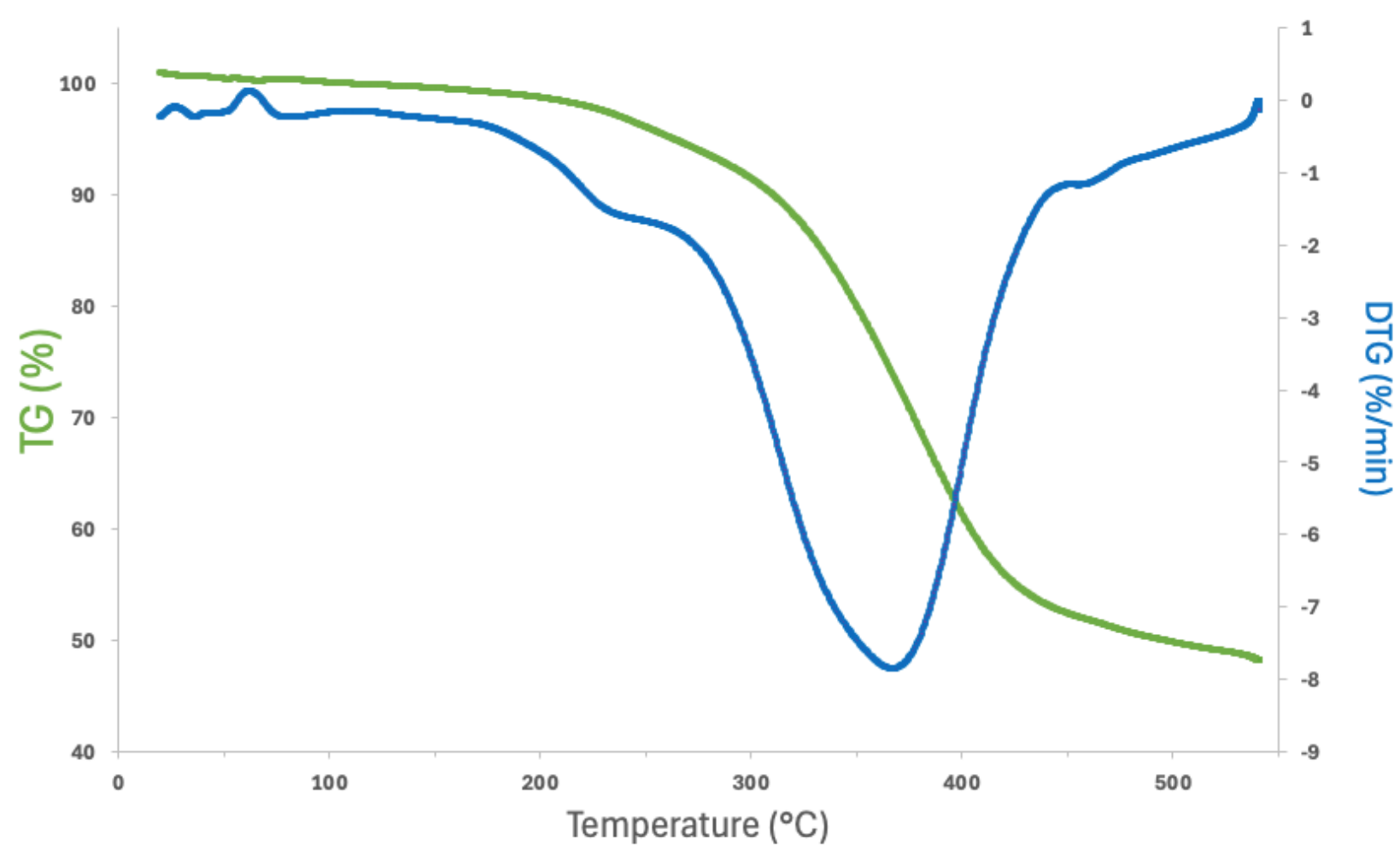

Figure ESI 38 – TGA of GTE grafted on Kraft lignin
